# 1,2,3-Triazolyl-tetrahydropyrimidine Conjugates as Potential Sterol Carrier Protein-2 Inhibitors: Larvicidal Activity against the Malaria Vector *Anopheles arabiensis* and In Silico Molecular Docking Study

**DOI:** 10.3390/molecules27092676

**Published:** 2022-04-21

**Authors:** Katharigatta N. Venugopala, Pottathil Shinu, Christophe Tratrat, Pran Kishore Deb, Raquel M. Gleiser, Sandeep Chandrashekharappa, Deepak Chopra, Mahesh Attimarad, Anroop B. Nair, Nagaraja Sreeharsha, Fawzi M. Mahomoodally, Michelyne Haroun, Mahmoud Kandeel, Syed Mohammed Basheeruddin Asdaq, Viresh Mohanlall, Nizar A. Al-Shar’i, Mohamed A. Morsy

**Affiliations:** 1 Department of Pharmaceutical Sciences, College of Clinical Pharmacy, King Faisal University, Al-Ahsa 31982, Saudi Arabia; ctratrat@kfu.edu.sa (C.T.); mattimarad@kfu.edu.sa (M.A.); anair@kfu.edu.sa (A.B.N.); sharsha@kfu.edu.sa (N.S.); mharoun@kfu.edu.sa (M.H.); momorsy@kfu.edu.sa (M.A.M.); 2 Department of Biotechnology and Food Science, Faculty of Applied Sciences, Durban University of Technology, Durban 4001, South Africa; vireshm@dut.ac.za; 3 Department of Biomedical Sciences, College of Clinical Pharmacy, King Faisal University, Al-Ahsa 31982, Saudi Arabia; spottathail@kfu.edu.sa; 4 Department of Pharmaceutical Sciences, Faculty of Pharmacy, Philadelphia University, Amman 19392, Jordan; 5 CREAN-IMBIV (UNC-CONICET), Av. Valparaiso s.n. and FCEFyN, Universidad Nacional de Cordoba, AV. Sarsfield 299, Cordoba 5000, Argentina; raquel.gleiser@unc.edu.ar; 6 Department of Medicinal Chemistry, National Institute of Pharmaceutical Education and Research (NIPER-R) Raebareli, Lucknow, UP 226002, India; c.sandeep@niperraebareli.edu.in; 7 Department of Chemistry, Indian Institute of Science Education and Research Bhopal, Bhopal By-Pass Road, Bhauri, Bhopal, MP 462066, India; dchopra@iiserb.ac.in; 8 Department of Pharmaceutics, Vidya Siri College of Pharmacy, Off Sarjapura Road, Bangalore, KA 560035, India; 9 Department of Health Sciences, Faculty of Medicine and Health Science, University of Mauritius, Réduit 80837, Mauritius; f.mahomoodally@uom.ac.mu; 10 Department of Biomedical Sciences, College of Veterinary Medicine, King Faisal University, Al-Ahsa 31982, Saudi Arabia; mkandeel@kfu.edu.sa; 11 Department of Pharmacology, Faculty of Veterinary Medicine, Kafrelsheikh University, Kafrelsheikh 33516, Egypt; 12 Department of Pharmacy Practice, College of Pharmacy, AlMaarefa University, Dariyah, Riyadh 13713, Saudi Arabia; sasdag@mcst.edu.sa; 13 Department of Medicinal Chemistry and Pharmacognosy, Faculty of Pharmacy, Jordan University of Science and Technology, Irbid 22110, Jordan; nashari@just.edu.jo; 14 Department of Pharmacology, Faculty of Medicine, Minia University, El-Minia 61511, Egypt

**Keywords:** mosquito larvicidal activity, *Anopheles arabiensis*, sterol carrier protein-2, molecular hybridization, triazole, pyrimidine, Biginelli reaction, click chemistry, in silico docking

## Abstract

Alteration of insect growth regulators by the action of inhibitors is becoming an attractive strategy to combat disease-transmitting insects. In the present study, we investigated the larvicidal effect of 1,2,3-triazolyl-pyrimidinone derivatives against the larvae of the mosquito *Anopheles arabiensis*, a vector of malaria. All compounds demonstrated insecticidal activity against mosquito larvae in a dose-dependent fashion. A preliminary study of the structure–activity relationship indicated that the electron-withdrawing substituent in the *para* position of the 4-phenyl-pyrimidinone moiety enhanced the molecules’ potency. A docking study of these derivatives revealed favorable binding affinity for the sterol carrier protein-2 receptor, a protein present in the intestine of the mosquito larvae. Being effective insecticides against the malaria-transmitting *Anopheles arabiensis*, 1,2,3-triazole-based pyrimidinones represent a starting point to develop novel inhibitors of insect growth regulators.

## 1. Introduction

**Figure 1:**
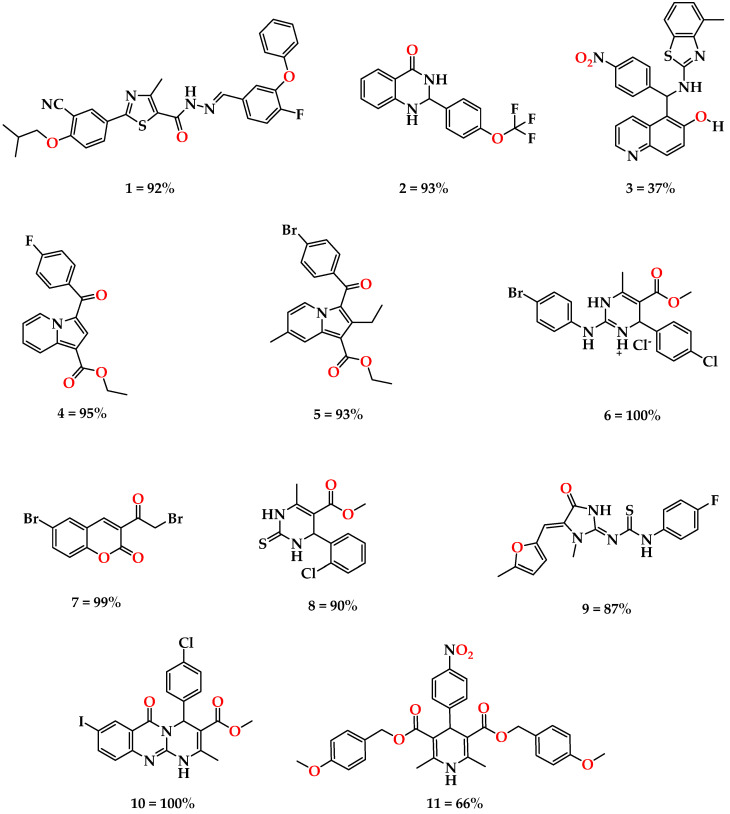
Various heterocyclic compounds promising as larvicidal agents, with their percentage inhibition.

According to the 2018 WHO Malaria report, 219 million people were affected by malaria in 2017. Despite the great progress made over the last decade, the decline of malaria cases has stalled and even reversed in some regions since 2014. Among the many challenges to be faced, threats to progress in malaria research include the continued emergence of parasite resistance to anti-malarial drugs and mosquito resistance to insecticides [1]. Vector control is an integral part of the management of diseases from insects. *Anopheles* female mosquitoes are mainly responsible for spreading malaria to humans in sub-Saharan Africa. To effectively reduce the transmissibility of malaria, four main classes of synthetic insecticides have been developed and are employed for indoor residual spraying against adult mosquitoes; they are organophosphates, carbamates, organochlorides, and pyrethroids [2]. These insecticides share similar actions as they disrupt the nervous system of the insect. Specifically, voltage-gated sodium channels [3] and acetylcholinesterase [4] are the two main molecular targets of the current insecticides controlling the malaria mosquito vector. However, their use presents neurotoxic effects in the human population, with the exception of pyrethroid insecticides, which are considered the safest insecticides for the control of malarial adult mosquitoes. Their lack of toxicity is due to their higher selectivity for insect sodium channels in comparison with human sodium channels [4]. However, the extensive use of insecticides has increasingly led to mosquito resistance, which threatens the vector control of disease-transmitting insects. Therefore, it is extremely important to explore new molecules with a different mode of action and less toxicity compared to the current insecticides so to develop an efficient vector control. Any molecule with the ability to interfere with the developmental stages of the insects from egg to pupa is considered a candidate to block disease transmission. Indeed, targeting insect growth is a new effective strategy to control the vector and appears to be less harmful than other strategies to the human population [5]. For instance, some insecticidal derivatives targeting mosquito larvae have been developed as inhibitors of juvenile hormone [6], calcium-dependent protein kinase-1 [7], sterol carrier protein-2 (SCP-2) [8], D7r4 [9], and purine nucleoside phosphorylase [10]. During the last decade, our research group has been interested in developing heterocyclic compounds with potential larvicidal properties against mosquito larvae of *Anopheles arabiensis*. We have identified a number of various heterocyclic compounds exhibiting such activity, including (*E*)-2-(3-cyano-4-isobutoxyphenyl)-*N*’-(4-fluoro-3-phenoxybenzylidene)-4-methylthiazole-5-carbohydrazide (**1**) [11], 2-(4-(trifluoromethoxy)phenyl)-2,3-dihydroquinazolin-4(1*H*)-one (**2**) [12], 5-(((4-methylbenzo[d]thiazol-2-yl)amino)(4-nitrophenyl)methyl)quinolin-6-ol (**3**) [13], ethyl 3-(4-fluorobenzoyl)indolizine-1-carboxylate (**4**) [14], ethyl 3-(4-bromobenzoyl)-2-ethyl-7-methylindolizine-1-carboxylate (**5**) [15], 2-(4-bromophenylamino)-6-(4-chlorophenyl)-5-(methoxycarbonyl)-4-methyl-3,6-dihydropyrimidin-1-ium chloride (**6**) [16], 6-bromo-3-(2-bromoacetyl)-2H-chromen-2-one (**7**) [17], methyl 4-(2-chlorophenyl)-6-methyl-2-thioxo-1,2,3,4-tetrahydropyrimidine-5-carboxylate (**8**) [18], 1-(4-fluorophenyl)-3-{(2*E*,5*Z*)-1-methyl-5-[(5-methylfuran-2-yl)methylidene]-4-oxoimidazolidin-2-ylidene}thiourea (**9**) [19], methyl 4-(4-chlorophenyl)-8-iodo-2-methyl-6-oxo-1,6-dihydro-4*H*-pyrimido [2,1-b]quinazoline-3-carboxylate (**10**) [20], and bis(4-methoxybenzyl) 2,6-dimethyl-4-(4-nitrophenyl)-1,4-dihydropyridine-3,5-dicarboxylate (**11**) [21] (Figure 1).

In the last few years, molecular hybridization has emerged as an attractive approach in drug discovery [22,23,24,25,26]. This approach is based on covalently linking two or more known bioactive pharmacophores to create a single unique molecular architecture scaffold. The hybrid molecule concept led to the development of multi-targeting compounds and may resolve the problem of drug resistance [27,28,29]. In this study, we explored the design of potentially active compounds by merging two well-known pharmacophores, i.e., dihydropyrimidine and triazole.

Dihydropyrimidine pharmacophores exhibit a wide spectrum of pharmacological activities such as anticancer [30], antiviral [31], antihypertensive [32], calcium channel blocking [33], anti-tubercular [34], antimicrobial [35], anti-inflammatory [36], and larvicidal actions [37]. Anti-malarial drugs such as halogenated dihydropyrimidine analogues [38] and pyrimethamine as a commercially available folic acid antagonist are used for the treatment and prevention of malaria or in combination with a sulfonamide to treat toxoplasmosis. The biological activity of dihydropyrimidines may depend on their interaction with lipophilic molecules on biological membranes, which can modulate the organization of the membranes and the functionality of their proteins, as suggested by studies with artificial membranes [39].

**Figure 2:**
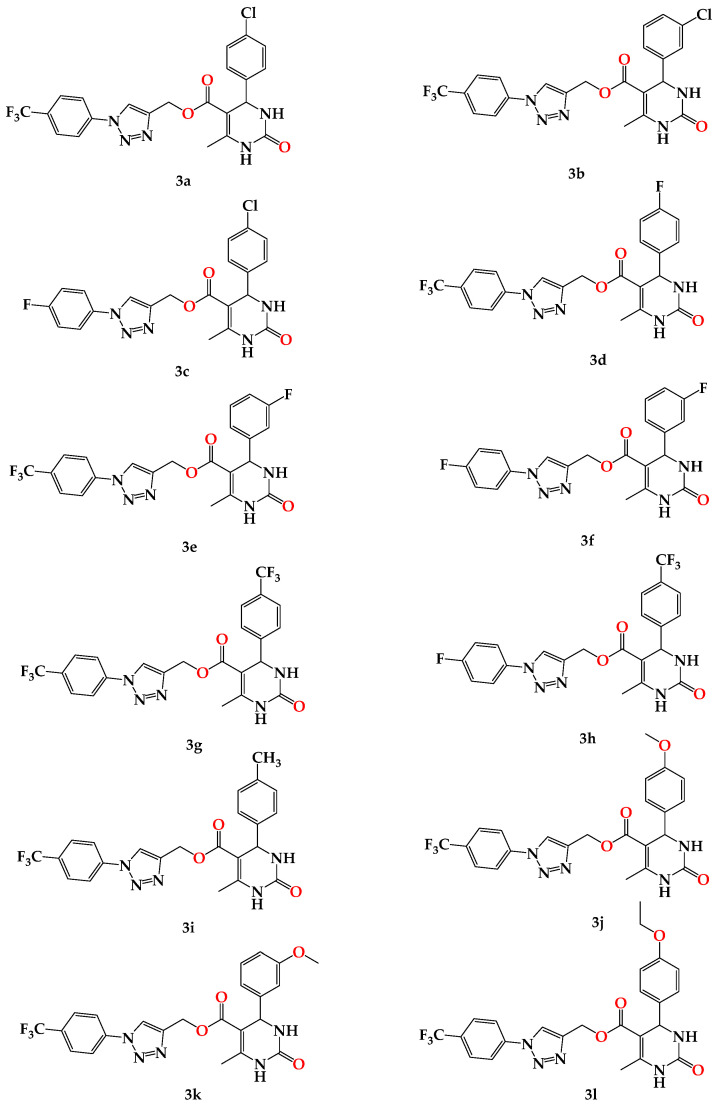
Chemical structures of 1,2,3-triazole-tetrahydropyrimidinone hybrids 3**a**–**l** tested for larvicidal activity against *Anopheles arabiensis*.

Triazoles are privileged pharmacophore scaffolds in drug discovery owing to their wide range of biological activities, such as anti-malaria [40], antimicrobial [41,42], antifungal [43,44], anticancer [45], antidiabetic [46], anti-inflammatory [47], antiviral [48], anti-tuberculosis [49,50], and Alzheimer’s disease inhibiting activities [51]. Several reviews highlighted the biological properties of these scaffolds [45,52,53,54]. To the best of our knowledge, there is only one report related to the molecular hybridization strategy involving the two pharmacophores 1,2,3-triazole and tetrahydropyrimidine for the development of potent DPP-4 inhibitors for type II diabetes mellitus treatment [55]. Recently, we exploited the success of the molecular hybridization strategy and combined the two pharmacophores mentioned above to generate 1,2,3-triazole-based tetrahydropyrimidinone scaffolds **3a–l** which displayed potent anti-tuberculosis activity (Figure 2) [49]. With these compounds in hand, we decided to investigate their larvicidal effect against the malaria vector *Anopheles arabiensis* and to explore their molecular target via in silico docking simulation.

## 2. Results and Discussion

### 2.1. Chemistry

**Scheme 1:**
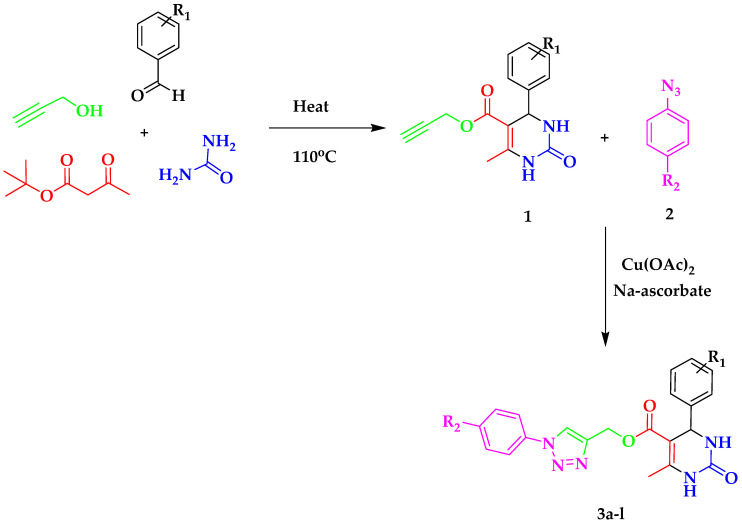
Synthetic construction of 1,2,3-triazole-tetrahydropyrimidinone hybrids **3a–l**.

Our previous research regarding the synthesis of tetrahydropyrimidinone 1,2,3-triazole-based conjugates **3a–l** showed interesting anti-*Mycobacterium tuberculosis* activity against susceptible and multi-drug resistant strains [49]. In this study, we wondered whether the hybrids **3a–l** could be used as insecticides against mosquito larvae of *Anopheles arabiensis* for blocking malaria transmission. The synthesis of the title compounds was achieved as outlined in Scheme 1. The hybrids **3a–l** were easily accessible in two steps by applying the Biginelli reaction followed by click chemistry.

### 2.2. Larvicidal Activity

**Figure 3:**
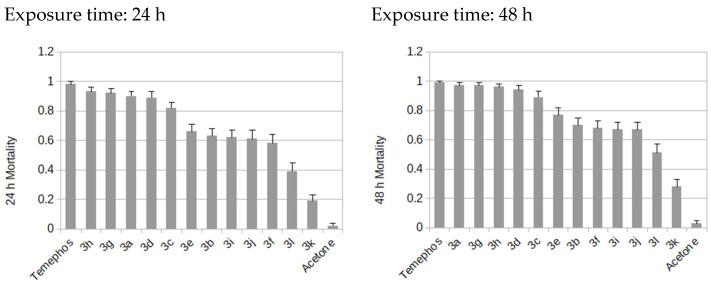
Average mortality of *Anopheles arabiensis* larvae exposed to the test compounds **3a–l**, Temephos at 4 µg/mL, and acetone (negative control), for 24 h and 48 h.

To study the potential larvicidal action of the 1,2,3-triazole-pyrimidinone conjugates **3a–l**, the mortality of third instar mosquito larvae of *Anopheles arabiensis* was monitored. The mortality of the larvae significantly differed (*p* < 0.0001) depending on the used compound, the exposure time, and the compound concentration. A dose-dependent effect of the larvicidal activity was observed with the tested hybrids; a relationship with the exposure time was also observed. The overall mortality increased from 10% at 1 µg/mL to 74% at 4 µg/mL, and from 24 h (32 ± 1%) to 48 h of exposure (38 ± 1%). Among the tested derivatives, **3g**, and **3h** were found to exert the highest larvicidal effect by producing more than 90% mosquito larvae mortality within 24 h of exposure. After 48 h of exposure at 4 µg/mL, mortality from exposure to compounds **3a**, **3d**, **3g**, and **3h** was statistically equivalent to that observed after exposure to the positive control Temephos (1mg/mL), while the compounds **3k** and **3l** showed the lowest larvicidal effect, with 28% and 51% mortality, respectively (Table 1, Figure 3).

Figure 3 (Table 1) shows the 24 h and 48 h mortality rate of larvae exposed to decreasing compound concentrations, starting at 4 µg/mL. Mortalities following any of the treatments were significantly higher than that observed upon treatment with the negative (acetone) control, indicating that all the triazole-pyrimidinone hybrids are toxic against mosquitoes. Interestingly, the rate of mortality increased with the increase of the time of exposure.

Based on the bioactivity results, a preliminary structure–activity relationship (SAR) for the hybrids could be established. It can be observed that the nature and the position of the substituent in the molecule have an influence on the larvicidal action of the tested compounds. For the series of 4-trifluoromethyl-phenyltriazoles derivatives **3a**, **3b**, **3d**, **3e**, **3g**, **3i**, **3j**, **3k**, and **3l**, it can be noticed that the presence of electron-withdrawing substituents at the phenyl ring attached to the pyrimidinone fragment resulted in higher toxicity, as observed for compounds **3a**, **3d**, and **3g** as compared to the compounds containing electron-rich groups (**3i–3l**). Thus, the derivatives **3a** (4-Cl), **3d** (4-F), **3g** (4-CF_3_) demonstrated a higher mortality rate with respect to the derivatives **3i** (4-CH_3_), **3j** (4-OCH_3_), **3l** (4-OEt) (Figure 3, 24 h). In addition, the compounds **3a** (4-Cl), **3d** (4-F), and **3g** (4-CF_3_) showed similar larvicidal potency, indicating the importance of electron-withdrawing substituents at the *para* position of the 4-phenyl-pyrimidinones. Shifting the substituent from the *para* to the *meta* position of the phenyl ring attached to the pyrimidinone fragment resulted in a diminution of the activity (Figure 3, 24 h). For instance, the comparison of the larvicidal activity of the following couples **3a** (4-Cl)/**3b** (3-Cl), **3d** (4-F)/**3e** (3-F), and **3j** (4-OCH_3_)/**3k** (3-OCH_3_) clearly showed a reduction in larvicidal potency by 50% after 24 h exposure. In contrast, the replacement of the trifluoromethyl group by a fluorine atom in the phenyl-triazole moiety was also found to be favorable for the activity, as seen for the couples **3a** (4-Cl)/**3c** (4-Cl), **3e** (3-F)/**3f** (3-F), and **3g** (4-CF_3_)/**3h** (4-CF_3_), which exhibited comparable activities. Our SAR study indicated that the electronic effect of substituents at the *para* position of both aromatic rings plays an important role in influencing the larvicidal activity of the 1,2,3-triazole-pyrimidinone hybrids. Particularly, the presence of an electro-withdrawing substituent was found to favor the potency of these molecules.

The lethal concentrations and the effects of the exposure time were further assessed for compounds showing the highest mortality. The 50% lethal concentration (LC50) (F_4,12_= 5.89; *p* = 0.003) varied significantly depending on the tested compound and was the lowest for Temephos on average. The estimated lethal concentration significantly changed also from 24 to 48 h of exposure (F_1,12_ = 7.39; *p* = 0.01). After 48 h of exposure, the LC50 of compounds **3a**, **3g**, and **3h** were as low as that of Temephos (Table 2).

### 2.3. Molecular Docking

On the basis of the significant larvicidal activities exerted by the hybrids, we carried out a computational docking study aiming at identifying plausible molecular targets. First, we investigated whether the hybrids **3a–l** might exert a neurotoxic action by examining in silico their blood–brain barrier penetration using Accelrys Discovery Studio. The results indicated that all derivatives were predicted to poorly penetrate the central nervous system, suggesting that both voltage-gated sodium channels and acetylcholinesterase are unlikely to be the molecular targets responsible for their activity. Moreover, this finding also indicated that the hybrids could be considered as safe insecticides, with a minimal risk of neurotoxic effects in humans. Next, we turned our attention to examining the action targets associated with the insect growth regulator. We speculated that the presence of fluorine and trifluoromethyl substituents at the 4-position of the phenyltriazole core might greatly contribute to the bioactivity of our compounds through the participation of hydrogen and halogen to the binding. Therefore, all known mosquito molecular targets, i.e., juvenile hormone (pdb 5v13), D7r4 (pdb 2pql and 2eqeh), sterol carrier protein-2 SCP-2 (pdb 1zp4), transaminase (pdb 2ch2), and calcium-dependent protein kinase-1 (pdb 4jbv) were screened in a docking simulation investigation. We found that the docking of our compounds either failed or provided a positive docking score against the above molecular targets with the exception of SCP-2 and calcium-dependent protein kinase-1. However, we found that the substituents of the title compounds played a critical role only in the binding with SCP-2. The molecular docking study revealed that the inhibition of SCP-2 might be the plausible mechanism of action of the examined compounds. SCP-2 is a protein located in the larvae mosquito intestine, acting as a cholesterol transporter necessary for larval development [56]. All the compounds were found to accommodate very well within the active site of SCP-2, as demonstrated by their good to excellent binding energy as compared to the lipid native ligand shown in Table 3. In addition, a computational study revealed that the R-stereoisomers of the hybrids **3a–l** were predicted to have stronger affinity for the SCP-2 binding cavity than their corresponding S-stereoisomers. The correlation between the activity and the binding energy of the derivatives remains unclear. As seen in Table 3, the least potent molecules **3k** and **3l** displayed excellent docking scores. However, it was observed that the potency of all hybrids **3a–l** increased with the increase of the exposure time, including that of the least potent compounds. Based on the above observation, the lack of correlation between the potency and the docking energy is more likely related to the pharmacokinetics properties of the derivatives. 

**Figure 4:**
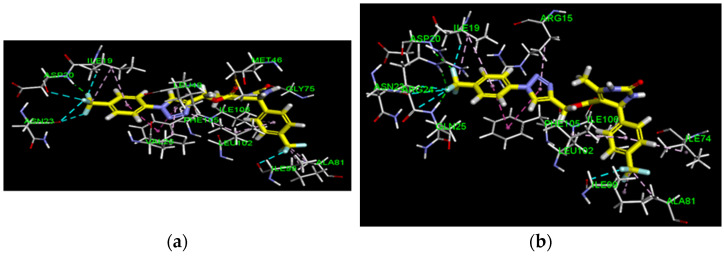
Predicted docking poses of the R- and S-stereoisomers of **3g** in the sterol carrier protein-2 binding domain (PDB 1pz4). H-bond, halogen (fluorine), pi–pi, and pi–alkyl interactions are represented as a dotted line in green, blue, violet, and grey, respectively. (**a**) **3g** R-form; (**b**) **3g** S-form.

The SCP-2 binding cavity has a unique structural feature, since the edge of the pocket is composed of the residues Ile99, Asp20, Asn23, Arg24, Gln25, and Val26, responsible for hydrogen bonding. The lipid native ligand interacts strongly with this region of the pocket through hydrogen bonding between its carboxylate ion and the amino acid residues Arg24, Gln25, and Val26. As for our compounds, it was predicted that the 4-trifluoromethyl and 4-fluorophenyltriazole fragment was located at the edge of the pocket, forming hydrogen bonding interactions with the residues Asp20, Arg24, and Gln25 and forming fluorine interactions with the residues Asp20 and Asn23 (Figure 4). Interestingly, the predicted binding mode of both stereoisomers for all compounds was observed to be similar to that of the binding of R- and S-stereoisomers of the hybrid **3g**, as depicted in Figure 4. The phenyltriazole core appeared oriented to the edge of the binding pocket, and the 4-phenylpyrimidinone moiety appeared located at the other side of the binding site where the phenyl group was accommodated into a sandwich between the amino acid residues Ala81 and Leu102 through hydrophobic interactions. An additional fluorine interaction at this site with the residue Ile99 was only observed for derivatives **3g** and **3h** having a 4-trifluoromethyl group from the phenylpyrimidinone moiety. Another major contribution to the activity is thought to be the formation of face-to-face pi–pi stacking hydrophobic interactions with the residue Phe105 and with both rings of the phenyltriazole moiety occurring for all compounds (Table 3, Figure 4) with the exception of 3i (4-CH_3_), whose triazole core is not involved in such interaction, which might be responsible for lowering the larvicidal activity (Figure 4). On the basis of SAR, the presence of an electron-withdrawing group in the para position is beneficial to the activity. The order of activity for 4-trifluorophenyltriazol derivatives with a substituent in the para position was as follows: 3g (4-CF_3_) > 3a (4-Cl) > 3d (4-F) > 3i (4-CH_3_) > 3j (4-OCH_3_) > 3l (4-OEt). However, at the molecular level, the hydrophobic character of a substituent rather than the electronic effect of the substituent appeared to be the key factor determining the larvicidal activity. Indeed, the substituents in the para position participated in hydrophobic interactions with two key amino acids, Ala 81 and Ile 99, with the exception of compound 3j (4-OCH_3_), which may explain its reduced activity (Figure 4). However, it is not clear why the derivative 3l (4-OEt), displaying such interactions, was the least potent. The size of the substituent may also be another factor contributing to the larvicidal properties. The docking simulation provided molecular insights into the SCP-2 binding domain and may serve as the starting point in the development of novel more potent congeners, thus helping confirm the mechanistic target as SCP-2.

### 2.4. Molecular Dynamics Simulations

**Figure 5:**
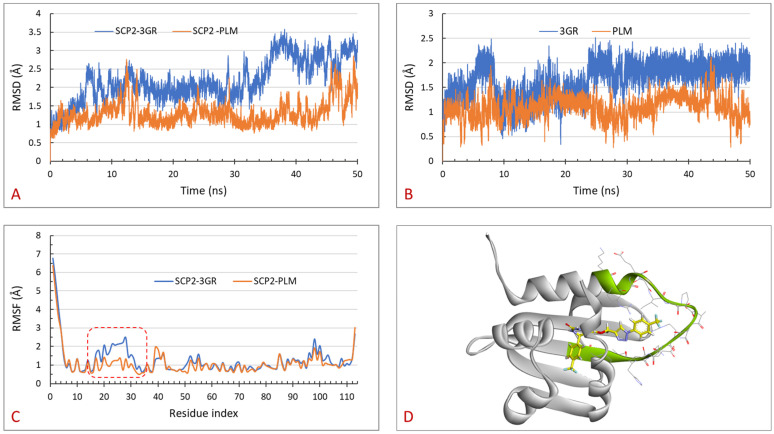
(**A**,**B**), RMSD plots for the simulated complexes (SCP2–3GR and SCP2–PLM) and the complexed ligands (3GR and PLM). (**C**), Represents the RMSF versus the residue index plot for the two simulated complexes. The protein segment of the SCP2–3GR complex that showed different (higher) flexibility compared to the SCP2–PLM crystal complex is highlighted by a dashed red rectangle and mapped onto the protein backbone in (**D**). The protein is shown as ribbon, 3GR in sticks with yellow carbons, and the highlighted segment in C is shown in green.

In order to further investigate the binding stability of the tested compounds bound to their identified putative target, we ran a 50 ns MD simulation for the SCP-2 virtual complex with one of the most active compounds (3gR). The top scoring pose of compound 3gR was used as a structural model for running the simulations. Moreover, for comparison purposes, we ran another 50 ns MD simulation for the crystal complex of SCP-2 with its native co-crystallized lipid (PLM) (PDB ID 1PZ4). The binding stability of the complexed ligands was investigated by tracking their dynamical behavior throughout the simulation time via calculating the root-mean-square deviations (RMSD) for the simulated complexes and the individual complexed ligands, in addition to their residual fluctuations (RMSF) (Figure 5).

**Figure 6:**
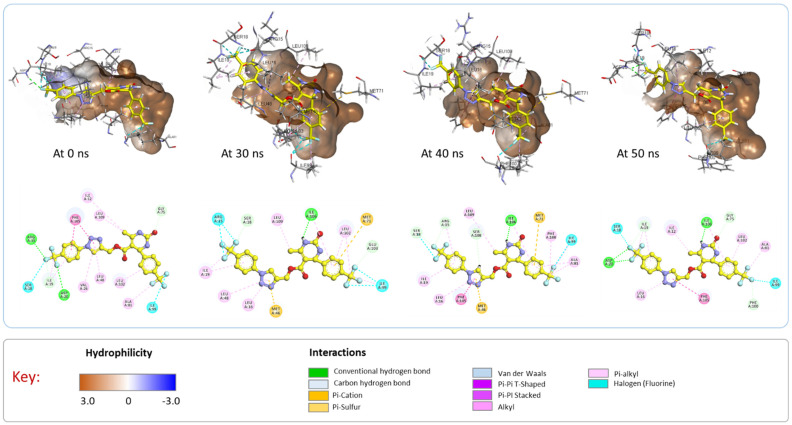
Snapshots of the simulated SCP2–3GR complex at different time points. Upper panel: 3D protein–ligand representations. The binding site is shown as a hydrophobic surface; 3GR is shown in balls and sticks, with carbons colored in yellow. Lower panel: 2D interaction maps of compound 3GR with residues’ binding sites. Residues are colored according to the type of their interactions with 3GR.

The average RMSD value for the SCP2–3GR complex was 2.17 Å, compared to 1.30 Å for the SCP2–PLM complex. The higher RMSD value for the virtual complex could be attributed to the dynamic induced fit that the complex experienced as it evolved with time to better accommodate the docked 3gR ligand and maximize the intermolecular interactions. Moreover, given the small size of the SCP-2 protein (113 aa) and the existence of a freely moving (loose) *N*-terminus (6 aa), this difference is less conclusive. On the other hand, a comparison of the RMSD values of the complexed ligands can give a better idea of their binding stability. The average RMSD value for compound 3gR was 1.67 Å, compared to 1.08 Å for the native lipid (PLM). This slight difference in RMSD values could be attributed to the induced fit mentioned above. Interestingly, during the last 25 ns of the simulation time, compound 3gR showed less RMSD fluctuations compared to the lipid, which is indicative of a stable binding. A visual inspection of the simulation trajectories (Figure 6, and Supplementary Material Movie S1) clearly showed that the 4-trifluoromethylphenyl part of compound 3gR flexed during the first 25 ns of the simulation time. Afterwards, it reached a stable conformation by establishing stable interactions with the amino acid residues Arg15, Ser18 (halogen bonds with CF_3_), and Ile19 (hydrophobic interactions with the phenyl ring) that were maintained until the end of the simulation time, in addition to intermittent hydrogen bonds with Asp20. The rest of compound 3gR (the (1-(4-(trifluoromethyl)phenyl)-1*H*-1,2,3-triazol-4-yl)methyl 6-methyl-2-oxo-1,2,3,4-tetrahydropyrimidine-5-carboxylate part) was well-anchored to the active site pocket throughout the simulation time. Moreover, the conformational flexibility of the simulated SCP2–3GR complex was also investigated by calculating the fitted residual fluctuations (RMSF) and by comparing it to the simulated SCP2–PLM crystal complex (Figure 5C,D). The average RMSF of the SCP2–3GR complex was 1.29 Å, compared to 1.12 Å for the SCP2–PLM complex. The segment of the SCP2–3GR complex that showed different (higher) flexibility compared to the SCP2–PLM crystal complex is highlighted with a dashed red rectangle in Figure 5C and mapped onto the protein backbone (Figure 5D). This segment corresponds to amino acid residues extending from Lys14 to Lys31 of the protein that include the loop that coordinates the carboxylate group of the fatty acid in the crystal complex (PDB ID 1PZ4) and interacts with the 4-trifluoromethylphenyl part of compound 3gR; this agrees with the above-mentioned dynamical induced-fit behavior of the SCP2–3GR complex.

In addition, the MMPBSA-based binding energy of SCP2 and of the compound 3GR was calculated based on the last 20 ns of the simulation trajectory and compared to that of the simulated crystal complex. The 3GR binding energy was −34.78 kcal/mol, compared to −39.42 kcal/mol for the SCP2–PLM complex. The close binding energy values indicate that compound 3GR has a good binding affinity towards the SPC2 protein. Collectively, the MD results support our findings that the SCP-2 protein is the likely putative target of the tested compounds.

## 3. Materials and Methods

### 3.1. Chemistry

The synthesis and the spectroscopy characterization data of the title compounds were previously reported by our research group [49].

### 3.2. Larvicidal Activity

The larvicidal screening was achieved as per the protocol described in our recent publication [12]. The vector *Anopheles arabiensis* employed was from a colonized strain from Zimbabwe, which had been reared according to the WHO (1975) guidelines [57] in an insectary, simulating the humidity (70%), lighting (12/12), and temperature (27.5 °C), of a malaria-endemic environment. One milliliter of a test compound at 1 mg/mL concentration was added to 250 mL of distilled water to produce a final concentration of 4 µg/mL; two further dilutions were prepared of 2 and 1 µg/mL. Thirty-third instar larvae were placed in a plastic container. Acetone and distilled water were used to set up a negative control, while the positive control was Temephos (Mostop; Agrivo), an effective emulsifiable organophosphate larvicidal used in the malaria control program. Each container was monitored for larval mortality at 24 h intervals for a period of two days and fed specially made cat food with reduced oil/fat content at regular intervals. Bioassays were performed three times. The percentage mortality was calculated relative to the initial number of exposed larvae. The LC50 was estimated using Dr. Alpha Raj Free LD50/LC50 Calculator (https://goo.gl/9QYcNk accessed on 3 March 2022), based on the method of Finney (1952) [58].

### 3.3. Data Analysis

First, the effects of test compound, dose, and exposure time on *Anopheles arabiensis* mortality were evaluated using generalized linear mixed models with quasi-binomial link function (GLMM; Infostat). The dependent variable was the number of dead larvae per container, the fixed effects were the compounds (**3a–l**, Temephos, acetone as the negative control), the dose (1, 2, or 4 µg/mL), and the exposure time (24 or 48 h). Next, the effects of compound and exposure time (24 or 48 h) on the LC50 of the most effective compounds were assessed using general linear models (Infostat). The dependent variable was the LC50, and the fixed effects were the compounds and the exposure time.

### 3.4. Molecular Modelling

The docking simulation was performed using the molecular modelling software Accelrys Discovery Studio Client 4.0. The X-ray crystal structure of the complex with a fatty acid inhibitor (1PZ4) was retrieved from the RSCB Protein Data Bank. The receptor preparation was accomplished by removing all ligands and water, adding hydrogen and the missing amino acid residues, and re-inserting the native ligand to the prepared crystal. To validate the docking protocol, the native ligand was subjected to re-docking to ensure proper orientation and interactions with the binding site. The C-Docker protocol was used to study key interactions of the title derivatives with the receptor. The ligands were docked into a rigid receptor, while a set of conformations were generated for each docked ligand. Additional scoring functions such as PLP1, PLP2, Jain, and PMF were further examined to ensure the optimal ligand orientation into the active binding site of the receptor. The highest negative (-ve) score of PLP1, PLP2, Jain, and PMF [25,59], indicating the strongest receptor–ligand binding affinities, was considered for refining the binding pose. The binding energy calculation was performed using the C-Docker protocol by selecting in situ ligand minimization.

### 3.5. Molecular Dynamics Simulations

The MD simulations were performed using AMBER 12 and the FF99SB force field [60]. The parameters for nonstandard residues (compound 3gR and PLM) were generated using the Antechamber program of Amber12, utilizing the General AMBER Force Field (GAFF) [61].

The same simulation protocol previously reported [62,63,64,65] was utilized in this study in which the simulated complexes were solvated using the TIP3P water model [66,67], sequentially minimized in three stages, gradually heated from 0 to 310 K over the course of 140 ps under constant volume conditions (NVT, the canonical ensemble), and equilibrated for 1 ns under constant pressure (NPT, the isothermal–isobaric ensemble); finally, the production phase under NPT conditions lasted for 50 ns. The SHAKE algorithm [68] was applied, and electrostatic interactions were treated using Particle Mesh Ewald with a non-bonded cut off value of 12 Å [69,70].

## 4. Conclusions

In summary, we presented the larvicidal activity of the 1,2,3-triazole-pyrimidine conjugates **3a–l** against the malaria vector *Anopheles arabiensis*. All the molecular hybrids demonstrated the ability to kill mosquito larvae in a dose-dependent manner. The LC50 concentration of the most promising compounds **3a**, **3g**, and **3h** was as low as that of Temephos, an organophosphate larvicide used for malaria control. A preliminary SAR study indicated that the presence of an electron-withdrawing substituent in the 4-position of the phenyl-pyrimidine moiety greatly enhanced the potency of the compounds. The molecular modelling study revealed SCP-2 as a plausible molecular target for the tested derivatives. Thus, these triazolyl-pyrimidine derivatives were found to be very effective, with larvae mosquitocidal properties and could serve as a template for developing a new and safer class of insecticides for vector control in the management of malaria.

## Figures and Tables

**Table 1:** Mortality of *Anopheles arabiensis* larvae exposed for 24 and 48 h to 1,2,3-triazolyl-tetrahydropyrimidine conjugates (**3a–l**) at 1 µg/mL, 2 µg/mL, and 4 µg/mL.

Compound Code	Mortality of *Anopheles Arabiensis* Larvae Exposed for 24 h			Mortality of *Anopheles Arabiensis* Larvae Exposed for 48 h		
	1 µg/mL *	2 µg/mL *	4 µg/mL *	1 µg/mL *	2 µg/mL *	4 µg/mL *
**3a**	0.19 ± 0.03 ^abc^	0.59 ± 0.04 ^de^	0.9 ± 0.03 ^fg^	0.21 ± 0.04 ^abcd^	0.64 ± 0.04 ^ef^	**0.97 ± 0.02 ^g^**
**3b**	0.13 ± 0.03 ^bchi^	0.33 ± 0.04 ^jk^	0.63 ± 0.04 ^e^	0.14 ± 0.03 ^dhi^	0.36 ± 0.04 ^jk^	0.70 ± 0.04 ^el^
**3c**	0.12 ± 0.03 ^chi^	0.42 ± 0.04 ^jl^	0.82 ± 0.03 ^gm^	0.17 ± 0.03 ^cdhi^	0.47 ± 0.04 ^jm^	0.89 ± 0.03 ^no^
**3d**	0.2 ± 0.03 ^abc^	0.58 ± 0.04 ^de^	0.89 ± 0.03 ^fg^	0.22 ± 0.04 ^abcd^	0.62 ± 0.04 ^efp^	0.94 ± 0.02 ^gn^
**3e**	0.07 ± 0.02 ^ino^	0.24 ± 0.04 ^ak^	0.66 ± 0.04 ^e^	0.09 ± 0.03 ^iqr^	0.29 ± 0.04 ^ak^	0.77 ± 0.04 ^ls^
**3f**	0.07 ± 0.02 ^ino^	0.21 ± 0.03 ^abc^	0.58 ± 0.04 ^de^	0.09 ± 0.03 ^iqr^	0.24 ± 0.04 ^abck^	0.68 ± 0.04 ^el^
**3g**	0.22 ± 0.04 ^ab^	0.5 ± 0.04 ^dl^	0.92 ± 0.02 ^fq^	0.24 ± 0.04 ^abck^	0.53 ± 0.04 ^fmp^	0.97 ± 0.02 ^g^
**3h**	0.17 ± 0.03 ^abch^	0.63 ± 0.04 ^e^	0.93 ± 0.02 ^fq^	0.21 ± 0.04 ^abcd^	0.69 ± 0.04 ^el^	0.96 ± 0.02 ^gn^
**3i**	0.08 ± 0.02 ^ino^	0.23 ± 0.04 ^ak^	0.62 ± 0.04 ^e^	0.10 ± 0.03 ^hiq^	0.26 ± 0.04 ^abck^	0.67 ± 0.04 ^el^
**3j**	0.09 ± 0.02 ^hio^	0.26 ± 0.04 ^ak^	0.61 ± 0.04 ^de^	0.11 ± 0.03 ^hiq^	0.28 ± 0.04 ^abk^	0.67 ± 0.04 ^el^
**3k**	0.03 ± 0.03 ^nop^	0.07 ± 0.02 ^ino^	0.19 ± 0.03 ^abc^	0.04 ± 0.02 ^qrt^	0.10 ± 0.03 ^hiq^	0.28 ± 0.04 ^abk^
**3l**	0.01 ± 0.01 ^p^	0.13 ± 0.03 ^bchi^	0.39 ± 0.04 ^jl^	0.02 ± 0.01 ^t^	0.18 ± 0.03 ^bcdh^	0.51 ± 0.04 ^mp^
**Acetone**	0.02 ± 0.01 ^np^	0.02 ± 0.01 ^np^	0.02 ± 0.01 ^np^	0.02 ± 0.01 ^t^	0.02 ± 0.01 ^t^	0.03 ± 0.02 ^rt^
**Temephos**	0.24 ± 0.04 ^ak^	0.78 ± 0.04 ^m^	0.98 ± 0.01 ^q^	0.27 ± 0.04 ^abck^	0.82 ± 0.03 ^os^	0.99 ± 0.01 ^g^

* ^a–t^ Compounds and/or concentrations for a given exposure time not sharing a letter are statistically different (*p* < 0.05). Mortalities not differing from the positive control Temephos are in bold.

**Table 2:** Comparison of the 50% lethal concentration (LC50) of selected compounds after 24 h and 48 h of exposure.

Compound Code	24 h LC50	48 h LC50	Mean LC50
**3d**	1.86 ± 0.08 ^a^	1.74 ± 0.08 ^a^	1.80 ± 0.06 ^a^
**3g**	1.81 ± 0.07 ^a^	1.66 ± 0.10 ^ab^	1.75 ± 0.06 ^a^
**3a**	1.85 ± 0.08 ^a^	1.65 ± 0.08 ^ab^	1.74 ± 0.06 ^a^
**3h**	1.80 ± 0.08 ^a^	1.63 ± 0.08 ^ab^	1.71 ± 0.06 ^a^
**Temephos**	1.48 ± 0.08 ^b^	1.43 ± 0.08 ^b^	1.46 ± 0.06 ^b^

^a, b^ LC50 values not sharing a letter are statistically different (*p* < 0.05).

**Table 3:** Binding energy and residues interaction of 1,2,3-triazole-pyrimidinone hybrids **3a–l** against sterol carrier protein-2 (PDB code: 1PZ4).

Entry	Stereoisomer	Binding Energy (kJ/mol)	Residues Interaction		
			H-Bond	Fluorine	Pi-Pi
**Native Ligand**	-	−96.64	Arg24, Gln25, Val26		
**3a**	*R*	−120.16	Asp20	Asp20, Asn23	Phe 105
	*S*	−88.81	Asp20	Asp20, Asn23	Phe 105
**3b**	*R*	−117.53	Asp20	Asp20, Asn23	Phe 105
	*S*	−119.95	Asp20, Arg24, Gln25	Asn23	Phe 105
**3c**	*R*	−119.82	Arg24, Gln25	Asn23	Phe 105
	*S*	−64.14	Arg24, Gln25	-	Phe 105
**3d**	*R*	−123.67	Asp20	Asp20, Asn23	Phe 105
	*S*	−119.40	Asp20, Arg24, Gln25	Asn23	Phe 105
**3e**	*R*	−120.87	Asp20	Asp20, Asn23	Phe 105
	*S*	−121.80	Asp20, Arg24, Gln25	Asn23	Phe 105
**3f**	*R*	−127.43	Arg24, Gln25	Asn23	Phe 105
	*S*	−105.08	Arg24, Gln25	-	Phe 105
**3g**	*R*	−118.60	Asp20	Asp20, Asn23, Ile99	Phe 105
	*S*	−72.04	Asp20, Arg24, Gln25	Asp20, Asn23, Ile99	Phe 105
**3h**	*R*	−127.47	Arg24, Gln25	Asp20, Arg24, Ile99	Phe 105
	*S*	−85.41	Arg24	Ile99	Phe 105
**3i**	*R*	−113.75	Arg15, Arg24	-	Phe 105
	*S*	−90.71	Arg24, Gln25	-	Phe 105
**3j**	*R*	−137.51	Asp20	Asp20, Asn23	Phe 105
	*S*	−121.20	Asp20, Arg24, Gln25	Asn23	Phe 105
**3k**	*R*	−131.34	Asp20, Arg24	Asp20, Asn23	Phe 105
	*S*	−116.49	Arg15, Asp20	-	Phe 105
**s**	*R*	−122.15	Asp20, Arg24	Asp20, Asn23	Phe 105
	*S*	−94.19	Gln25	-	Phe 105

## Data Availability

Not applicable.

## Supplementary Materials

The following supporting information can be downloaded at: https://www.mdpi.com/article/10.3390/molecules27092676/s1.

## Sample Availability

Samples of the compounds 3**a**–**l** are available from the authors.
