# Supplementary material for: 1,2,3-Triazolyl-tetrahydropyrimidine Conjugates as Potential Sterol Carrier Protein-2 Inhibitors: Larvicidal Activity against the Malaria Vector Anopheles arabiensis and In Silico Molecular Docking Study
Source: Molecules. 2022 Apr 21;27(9):2676. doi: 10.3390/molecules27092676 (PMC9102322; doi:10.3390/molecules27092676)
Supplement: Supplementary file 1 [file molecules-27-02676-s001.zip › molecules-1646621-SM.pdf]

1,2,3-Triazolyl-Tetrahydropyrimidine Conjugates as Potential Sterol Carrier Protein-2 Inhibitors: Larvicidal Activity Against Malaria Vector *Anopheles arabiensis* and *In Silico* Molecular Docking Study

Katharigatta N. Venugopala [1,2,*], Pottathil Shinu [3], Christophe Tratrat [1], Pran Kishore Deb [4*], Raquel M. Gleiser [5], Sandeep Chandrashekharappa [6], Deepak Chopra [7], Mahesh Attimarad [1], Anroop B. Nair [1], Nagaraja Sreeharsha [1,8], Fawzi M. Mahomoodally [9], Michelyne Haroun [1], Mahmoud Kandeel [10,11], Syed Mohammed Basheeruddin Asdaq [12], Viresh Mohanlall [2], Nizar A. Al-Shar'i [13], Mohamed A. Morsy [1,14]

[1] Department of Pharmaceutical Sciences, College of Clinical Pharmacy, King Faisal University, Al-Ahsa 31982, Saudi Arabia; ctratrat@kfu.edu.sa (C.T.); mattimarad@kfu.edu.sa (M.A.); anair@kfu.edu.sa (A.B.N.);
sharsha@kfu.edu.sa (N.S.); mharoun@kfu.edu.sa (M.H.); momorsy@kfu.edu.sa (M.A.M.)

[2] Department of Biotechnology and Food Science, Faculty of Applied Sciences, Durban University of Technology, Durban 4001, South Africa; vireshm@dut.ac.za

[3] Department of Biomedical Sciences, College of Clinical Pharmacy, King Faisal University, Al-Ahsa 31982, Saudi Arabia; spottathail@kfu.edu.sa (P.S.)

[4] Department of Pharmaceutical Sciences, Faculty of Pharmacy, Philadelphia University, Amman 19392, Jordan; prankishore1@gmail.com (P.K.D.)

[5] CREAN-IMBIV (UNC-CONICET), Av Valparaiso s.n., and FCEFyN, Universidad Nacional de Cordoba, Av .V. Sarsfield 299, Cordoba 5000, Argentina; raquel.gleiser@unc.edu.ar (R.M.G.)

[6] Department of Medicinal Chemistry, National Institute of Pharmaceutical Education and Research (NIPER-R) Raebareli, Lucknow UP 226002, India; c.sandeep@niperraebareli.edu.in (S.C.)

[7] Department of Chemistry, Indian Institute of Science Education and Research Bhopal, Bhopal By-pass Road, Bhauri, Bhopal 462066, Madhya Pradesh, India; dchopra@iiserb.ac.in (S.C.)

[8] Department of Pharmaceutics, Vidya Siri College of Pharmacy, Off Sarjapura Road, Bangalore 560035, India

[9] Department of Health Sciences, Faculty of Medicine and Health Science, University of Mauritius, Réduit, Mauritius; f.mahomoodally@uom.ac.mu (F.M.M.)

[10] Department of Biomedical Sciences, College of Veterinary Medicine, King Faisal University, Al-Ahsa 31982, Saudi Arabia; mkandeel@kfu.edu.sa (M.K.)

[11] Department of Pharmacology, Faculty of Veterinary Medicine, Kafrelsheikh University, Kafrelsheikh 33516, Egypt

[12] Department of Pharmacy Practice, College of Pharmacy, AlMaarefa University, Dariyah, Riyadh 13713, Saudi Arabia; sasdag@mcst.edu.sa (S.M.B.A.)

[13] Department of Medicinal Chemistry and Pharmacognosy, Faculty of Pharmacy, Jordan University of Science and Technology, P.O. Box 3030, Irbid, 22110, Jordan; nashari@just.edu.jo (N.A.A.)

[14] Department of Pharmacology, Faculty of Medicine, Minia University, El-Minia 61511, Egypt

* Correspondence: kvenugopala@kfu.edu.sa (K.N.V.); prankishore1@gmail.com (P.K.D.)

TABLE OF CONTENTS

Figures	Contents	Page number
	General Information	4
	General procedure for the synthesis of (1-(4-substitutedphenyl)-1*H*-1,2,3-triazol-4-yl)methyl 4-substitutedphenyl-6-methyl-2-oxo-1,2,3,4-tetrahydropyrimidine-5-carboxylates (**3a-l**)	4
**S1**	^{1}H-NMR of (1-(4-(trifluoromethyl)phenyl)-1*H*-1,2,3-triazol-4-yl)methyl 4-(4-chlorophenyl)-6-methyl-2-oxo-1,2,3,4-tetrahydropyrimidine-5-carboxylate (**3a**)	9
**S2**	^{13}C-NMR of (1-(4-(trifluoromethyl)phenyl)-1*H*-1,2,3-triazol-4-yl)methyl 4-(4-chlorophenyl)-6-methyl-2-oxo-1,2,3,4-tetrahydropyrimidine-5-carboxylate **(3a)**	10
**S3**	^{1}H-NMR of (1-(4-(trifluoromethyl)phenyl)-1*H*-1,2,3-triazol-4-yl)methyl 4-(3-chlorophenyl)-6-methyl-2-oxo-1,2,3,4-tetrahydropyrimidine-5-carboxylate (**3b**)	11
**S4**	^{13}C-NMR of (1-(4-(trifluoromethyl)phenyl)-1*H*-1,2,3-triazol-4-yl)methyl 4-(3-chlorophenyl)-6-methyl-2-oxo-1,2,3,4-tetrahydropyrimidine-5-carboxylate (**3b**)	12
**S5**	^{1}H-NMR of(1-(4-fluorophenyl)-1*H*-1,2,3-triazol-4-yl)methyl 4-(4-chlorophenyl)-6-methyl-2-oxo-1,2,3,4-tetrahydropyrimidine-5-carboxylate (**3c**)	13
**S6**	^{13}C-NMR of (1-(4-fluorophenyl)-1*H*-1,2,3-triazol-4-yl)methyl 4-(4-chlorophenyl)-6-methyl-2-oxo-1,2,3,4-tetrahydropyrimidine-5-carboxylate (**3c**)	14
**S7**	^{1}H-NMR of (1-(4-(trifluoromethyl)phenyl)-1*H*-1,2,3-triazol-4-yl)methyl 4-(4-fluorophenyl)-6-methyl-2-oxo-1,2,3,4-tetrahydropyrimidine-5-carboxylate (**3d**)	15
**S8**	^{13}C-NMR of (1-(4-(trifluoromethyl)phenyl)-1*H*-1,2,3-triazol-4-yl)methyl 4-(4-fluorophenyl)-6-methyl-2-oxo-1,2,3,4-tetrahydropyrimidine-5-carboxylate (**3d**)	16
**S9**	^{1}H-NMR of (1-(4-(trifluoromethyl)phenyl)-1*H*-1,2,3-triazol-4-yl)methyl 4-(3-fluorophenyl)-6-methyl-2-oxo-1,2,3,4-tetrahydropyrimidine-5-carboxylate (**3e**)	17
**S10**	^{13}C-NMR of (1-(4-(trifluoromethyl)phenyl)-1*H*-1,2,3-triazol-4-yl)methyl 4-(3-fluorophenyl)-6-methyl-2-oxo-1,2,3,4-tetrahydropyrimidine-5-carboxylate (**3e**)	18
**S11**	^{1}H-NMR of (1-(4-fluorophenyl)-1*H*-1,2,3-triazol-4-yl)methyl 4-(3-fluorophenyl)-6-methyl-2-oxo-1,2,3,4-tetrahydropyrimidine-5-carboxylate (**3f**)	19
**S12**	^{13}C-NMR of (1-(4-fluorophenyl)-1*H*-1,2,3-triazol-4-yl)methyl 4-(3-fluorophenyl)-6-methyl-2-oxo-1,2,3,4-tetrahydropyrimidine-5-carboxylate (**3f**)	20
**S13**	^{1}H-NMR of (1-(4-(trifluoromethyl)phenyl)-1*H*-1,2,3-triazol-4-yl)methyl 6-methyl-2-oxo-4-(4-(trifluoromethyl)phenyl)-1,2,3,4-tetrahydropyrimidine-5-carboxylate (**3g**)	21
**S14**	^{13}C-NMR of (1-(4-(trifluoromethyl)phenyl)-1*H*-1,2,3-triazol-4-yl)methyl 6-methyl-2-oxo-4-(4-(trifluoromethyl)phenyl)-1,2,3,4-tetrahydropyrimidine-5-carboxylate (**3g**)	22
**S15**	^{1}H-NMR of (1-(4-fluorophenyl)-1*H*-1,2,3-triazol-4-yl)methyl 6-methyl-2-oxo-4-(4-(trifluoromethyl)phenyl)-1,2,3,4-tetrahydropyrimidine-5-carboxylate (**3h**)	23

**S16**	^{13}C-NMR of (1-(4-fluorophenyl)-1*H*-1,2,3-triazol-4-yl)methyl 6-methyl-2-oxo-4-(4-(trifluoromethyl)phenyl)-1,2,3,4-tetrahydropyrimidine-5-carboxylate (**3h**)	24
**S17**	^{1}H-NMR of (1-(4-(trifluoromethyl)phenyl)-1*H*-1,2,3-triazol-4-yl)methyl 6-methyl-2-oxo-4-(*p*-tolyl)-1,2,3,4-tetrahydropyrimidine-5-carboxylate (**3i**)	25
**S18**	^{13}C-NMR of (1-(4-(trifluoromethyl)phenyl)-1*H*-1,2,3-triazol-4-yl)methyl 6-methyl-2-oxo-4-(*p*-tolyl)-1,2,3,4-tetrahydropyrimidine-5-carboxylate (**3i**)	26
**S19**	^{1}H-NMR of (1-(4-(trifluoromethyl)phenyl)-1*H*-1,2,3-triazol-4-yl)methyl 4-(4-methoxyphenyl)-6-methyl-2-oxo-1,2,3,4-tetrahydropyrimidine-5-carboxylate (**3j**)	27
**S20**	^{13}C-NMR of (1-(4-(trifluoromethyl)phenyl)-1*H*-1,2,3-triazol-4-yl)methyl 4-(4-methoxyphenyl)-6-methyl-2-oxo-1,2,3,4-tetrahydropyrimidine-5-carboxylate (**3j**)	28
**S21**	^{1}H-NMR of(1-(4-(trifluoromethyl)phenyl)-1*H*-1,2,3-triazol-4-yl)methyl 4-(3-methoxyphenyl)-6-methyl-2-oxo-1,2,3,4-tetrahydropyrimidine-5-carboxylate (**3k**)	29
**S22**	^{13}C-NMR of (1-(4-(trifluoromethyl)phenyl)-1*H*-1,2,3-triazol-4-yl)methyl 4-(3-methoxyphenyl)-6-methyl-2-oxo-1,2,3,4-tetrahydropyrimidine-5-carboxylate (**3k**)	30
**S23**	^{1}H-NMR of (1-(4-(trifluoromethyl)phenyl)-1*H*-1,2,3-triazol-4-yl)methyl 4-(4-ethoxyphenyl)-6-methyl-2-oxo-1,2,3,4-tetrahydropyrimidine-5-carboxylate (**3l**)	31
**S24**	^{13}C-NMR of (1-(4-(trifluoromethyl)phenyl)-1*H*-1,2,3-triazol-4-yl)methyl 4-(4-ethoxyphenyl)-6-methyl-2-oxo-1,2,3,4-tetrahydropyrimidine-5-carboxylate (**3l**)	32

1. General Information

All the chemicals were purchased from Sigma-Aldrich Corporation (analytical grade) and were used without further purification. FT-IR spectra were registered on a Bruker IFS 55 equinox FTIR spectrophotometer as KBr discs. ^{1}H- and ^{13}C-NMR spectra were recorded using a Bruker 400 or 500 MHz spectrometer in the solvents indicated (referenced to the residual 1H signals in the deuterated solvents) using TMS as internal standard. Chemical shifts are reported in ppm (δ scale), coupling constant (*J*) values are given in hertz (Hz). The splitting patterns are abbreviated as follows: s, singlet; d, doublet; m, multiplet. TLC analysis of reaction mixtures was performed on Merck aluminium plates coated with silica gel (60 F254). Compounds were visualized by ultra-violet irradiation at 254 and 366nm. Merck silica gel (60-120 mesh) was used for column chromatography.

2. General procedure for the synthesis of (1-(4-substitutedphenyl)-1H-1,2,3-triazol-4-yl)methyl 4-substitutedphenyl-6-methyl-2-oxo-1,2,3,4-tetrahydropyrimidine-5-carboxylates (3a-l)

A 25 ml round bottom flask equipped with a condenser was charged with substituted arylazide (1.0 mmol) as well as DHPMs having terminal alkynyl group (1.0 mmol) which was synthesized by the four component Biginelli like cyclo-condensation reaction of *tert*-butyl β-ketoester (1.0 mmol), propargyl alcohol (1.2 mmol), arylaldehyde (1.0 mmol) and urea (1.2 mmol). The entire reaction mixture allowed to stir for 3h at room temperature along with catalytic amount of $Cu(OAc)_2$ (0.1 mmol) and sodium ascorbate (0.2 mmol) in 1 : 2 ratio of acetone and water (2 ml) as a solvent till the reaction was complete. The progress of the reaction was monitored by TLC (4: 6 of Hexane and Ethylacetate). After completion of the reaction as indicated on TLC, the contents were concentrated under reduced pressure to remove excess of acetone and the crude reaction mixture was extracted with ethyl acetate and water. The combined organic extract, after drying over

anhydrous sodium sulfate, was again concentrated under reduced pressure to obtain the product. For analytically pure products, the final solid mass was purified by column chromatography using the Hexane/EtOAc (4: 6) as the eluent to give the pure product in 77 – 93 % yield.

2.1. (1-(4-(Trifluoromethyl)phenyl)-1*H*-1,2,3-triazol-4-yl)methyl 4-(4-chlorophenyl)-6-methyl-2-oxo-1,2,3,4-tetrahydropyrimidine-5-carboxylate (3a)

IR (KBr) ν/cm^{-1} 3365, 2966, 2309, 1709, 1638, 1331, 843, 786, 746. ^{1}H NMR (400 MHz, DMSO) δ 9.30 (s, 1H), 8.75 (s, 1H), 8.11 (d, *J* = 8.5 Hz, 2H), 7.96 (d, *J* = 8.6 Hz, 2H), 7.76 (s, 1H), 7.27 (d, *J* = 8.4 Hz, 2H), 7.17 (d, *J* = 8.4 Hz, 2H), 5.31 – 5.03 (m, 3H), 2.24 (s, 3H). ^{13}C NMR (101 MHz, DMSO) δ 165.2, 152.2, 150.2, 144.3, 144.0, 139.7, 132.2, 129.4, 128.7, 128.6, 127.7, 127.6, 123.2, 121.0, 98.6, 56.7, 53.8, 18.4. LCMS: 491.2 (M$^+$), 493.2 (M+2).

2.2. (1-(4-(Trifluoromethyl)phenyl)-1*H*-1,2,3-triazol-4-yl)methyl 4-(3-chlorophenyl)-6-methyl-2-oxo-1,2,3,4-tetrahydropyrimidine-5-carboxylate (3b)

IR (KBr) ν/cm^{-1} 3363, 2969, 2349, 1695, 1330, 843, 788, 757. ^{1}H NMR (400 MHz, DMSO) δ 9.33 (s, 1H), 8.80 (s, 1H), 8.11 (d, *J* = 8.4 Hz, 2H), 7.96 (d, *J* = 8.5 Hz, 2H), 7.79 (s, 1H), 7.34 – 7.13 (m, 4H), 5.20 – 5.13 (m, 3H), 2.25 (s, 3H). ^{13}C NMR (101 MHz, DMSO) δ 165.2, 158.0, 152.2, 150.5, 147.5, 146.9, 144.2, 133.3, 130.8, 127.7, 127.6, 126.6, 125.2, 123.2, 121.0, 98.4, 56.7, 53.9, 18.4. LCMS: 491.2 (M$^+$), 493.2 (M+2).

2.3. (1-(4-Fluorophenyl)-1*H*-1,2,3-triazol-4-yl)methyl 4-(4-chlorophenyl)-6-methyl-2-oxo-1,2,3,4-tetrahydropyrimidine-5-carboxylate (3c)

IR (KBr) ν/cm^{-1} 3414, 2960, 2349, 1712, 1640, 1323, 838, 792, 710. ^{1}H NMR (400 MHz, DMSO) δ 9.36 (s, 1H), 8.63 (s, 1H), 7.93 – 7.79 (m, 3H), 7.59 (d, *J* = 7.9 Hz, 2H), 7.45 – 7.38 (m, 4H), 5.27 – 5.09 (m, 3H), 2.25 (s, 3H). ^{13}C NMR (101 MHz, DMSO) δ 165.2, 152.2, 150.5, 149.5, 143.8, 127.5, 125.8, 125.8, 123.2, 122.8, 122.8, 117.3, 117.0, 98.4, 56.8, 54.1, 31.1, 23.1, 18.4. LCMS: 441.2 (M$^+$), 443.2 (M+2).

2.4. (1-(4-(Trifluoromethyl)phenyl)-1*H*-1,2,3-triazol-4-yl)methyl 4-(4-fluorophenyl)-6-methyl-2-oxo-1,2,3,4-tetrahydropyrimidine-5-carboxylate (3d)

IR (KBr) ν/cm^{-1} 3333, 2946, 2370, 1670, 1332, 844, 785, 753. ^{1}H NMR (500 MHz, DMSO) δ 9.29 (s, 1H), 8.72 (s, 1H), 8.12 (d, *J* = 8.5 Hz, 2H), 7.99 (d, *J* = 8.6 Hz, 2H), 7.77 (s, 1H), 7.24 – 7.21 (m, 2H), 7.06 (t, *J* = 8.8 Hz, 2H), 5.21 – 5.17 (m, 3H), 2.27 (s, 3H). ^{13}C NMR (126 MHz, DMSO) δ 165.3, 162.7, 160.7, 152.3, 150.04, 144.2, 141.3, 141.2, 139.6, 128.7, 128.6, 127.6, 123.2, 121.0, 115.6, 115.4, 99.0, 56.6, 53.7, 18.3. LCMS: 475.2.

2.5. (1-(4-(Trifluoromethyl)phenyl)-1*H*-1,2,3-triazol-4-yl)methyl 4-(3-fluorophenyl)-6-methyl-2-oxo-1,2,3,4-tetrahydropyrimidine-5-carboxylate (3e)

IR (KBr) ν/cm^{-1} 3367, 2979, 2345, 1700, 1636, 1329, 873, 845, 757. ^{1}H NMR (400 MHz, DMSO) δ 9.31 (s, 1H), 8.80 (s, 1H), 8.10 (d, *J* = 7.9 Hz, 2H), 7.96 (d, *J* = 8.0 Hz, 2H), 7.79 (s, 1H), 7.27 (d, *J* = 6.6 Hz, 1H), 7.06 – 6.87 (m, 3H), 5.27 – 5.10 (m, 3H), 2.24 (s, 3H). ^{13}C NMR (101 MHz, DMSO) δ 165.2, 163.6, 161.2, 152.3, 150.4, 147.9, 144.2, 139.7, 130.9, 130.8, 127.7, 127.6, 123.2, 122.5, 121.0, 114.6, 114.4, 113.5, 113.3, 98.5, 56.7, 53.8, 18.4. LCMS: 475.4.

2.6. (1-(4-Fluorophenyl)-1*H*-1,2,3-triazol-4-yl)methyl 4-(3-fluorophenyl)-6-methyl-2-oxo-1,2,3,4-tetrahydropyrimidine-5-carboxylate (3f)

IR (KBr) ν/cm^{-1} 3324, 3079, 2349, 1663, 1638, 1239, 845, 761, 703. ^{1}H NMR (400 MHz, DMSO) δ 9.31 (s, 1H), 8.62 (s, 1H), 7.90 –7.87 (m, 2H), 7.79 (s, 1H), 7.43 (t, *J* = 8.7 Hz, 2H), 7.28 (m, 1H), 7.06 – 6.86 (m, 3H), 5.23 – 5.10 (m, 3H), 2.25 (s, 3H). ^{13}C NMR (101 MHz, DMSO) δ 165.2, 163.7, 161.2, 160.9, 152.3, 150.4, 147.9, 147.9, 143.8, 133.5, 130.9, 130.8, 123.2, 123.0, 122.9, 122.5, 117.3, 117.0, 114.6, 114.4, 113.5, 113.3, 98.5, 56.8, 53.8, 18.4. LCMS: 425.4.

2.7. (1-(4-(Trifluoromethyl)phenyl)-1*H*-1,2,3-triazol-4-yl)methyl 6-methyl-2-oxo-4-(4-(trifluoromethyl)phenyl)-1,2,3,4-tetrahydropyrimidine-5-carboxylate (3g)

IR (KBr) ν/cm^{-1} 3412, 2968, 2345, 1700, 1641, 1326, 844, 795, 718. ^{1}H NMR (400 MHz, DMSO) δ 9.35 (s, 1H), 8.79 (s, 1H), 8.10 (d, *J* = 8.1 Hz, 2H), 7.95 (d, *J* = 8.2 Hz, 2H), 7.83 (s, 1H), 7.57 (d, *J* = 7.7 Hz, 2H), 7.38 (d, *J* = 7.6 Hz, 2H), 5.25 – 5.12 (m, 3H), 2.25 (s, 3H). ^{13}C NMR (101 MHz, DMSO) δ 165.1, 152.2, 150.5, 144.2, 139.6, 127.6, 127.6, 127.6, 127.5, 125.8, 125.7, 123.2, 120.9, 98.3, 59.3, 56.7, 54.1, 18.4. LCMS: 525.1.

2.8. (1-(4-Fluorophenyl)-1*H*-1,2,3-triazol-4-yl)methyl 6-methyl-2-oxo-4-(4-(trifluoromethyl)phenyl)-1,2,3,4-tetrahydropyrimidine-5-carboxylate (3h)

IR (KBr) ν/cm^{-1} 3367, 2960, 2349, 1711, 1638, 1313, 835, 787, 691. ^{1}H NMR (400 MHz, DMSO) δ 9.30 (s, 1H), 8.58 (s, 1H), 7.90 –7.87 (m, 2H), 7.76 (s, 1H), 7.43 (t, *J* = 8.4 Hz, 2H), 7.28 (d, *J* = 8.0 Hz, 2H), 7.18 (d, *J* = 8.0 Hz, 2H), 5.20 – 5.09 (m, 3H), 2.24 (s, 3H). ^{13}C NMR (101 MHz, DMSO) δ 165.2, 152.2, 150.2, 144.0, 143.9, 133.5, 132.2, 128.7, 128.6, 123.1, 123.0, 122.9, 98.6, 56.8, 53.8, 31.1, 18.4. LCMS: 475.3.

2.9. (1-(4-(Trifluoromethyl)phenyl)-1*H*-1,2,3-triazol-4-yl)methyl 6-methyl-2-oxo-4-(p-tolyl)-1,2,3,4-tetrahydropyrimidine-5-carboxylate (3i)

IR (KBr) ν/cm^{-1} 3367, 2967, 2349, 1710, 1646, 1324, 841, 792, 703. ^{1}H NMR (400 MHz, DMSO) δ 9.21 (s, 1H), 8.68 (s, 1H), 8.10 (d, *J* = 8.5 Hz, 2H), 7.97 (d, *J* = 8.6 Hz, 2H), 7.68 (s, 1H), 7.05 – 6.99 (m, 4H), 5.21 – 5.09 (m, 3H), 2.23 (s, 3H), 2.12 (s, 3H). ^{13}C NMR (101 MHz, DMSO) δ 165.3, 152.4, 149.7, 144.4, 142.2, 139.7, 136.8, 129.2, 127.7, 127.6, 126.6, 125.6, 123.1, 121.0, 99.2, 56.6, 54.0, 20.9, 18.3. LCMS: 471.2.

2.10. (1-(4-(Trifluoromethyl)phenyl)-1*H*-1,2,3-triazol-4-yl)methyl 4-(4-methoxyphenyl)-6-methyl-2-oxo-1,2,3,4-tetrahydropyrimidine-5-carboxylate (3j)

IR (KBr) ν/cm^{-1} 3390, 2964, 2345, 1695, 1638, 1336, 845, 790, 757. ^{1}H NMR (400 MHz, DMSO) δ 9.21 (s, 1H), 8.68 (s, 1H), 8.10 (d, *J* = 8.4 Hz, 2H), 7.96 (d, *J* = 8.6 Hz, 2H), 7.66 (s, 1H), 7.08 (d, *J* = 8.6 Hz, 2H), 6.76 (d, *J* = 8.6 Hz, 2H), 5.18 – 5.08 (m, 3H), 3.59 (s,

3H), 2.23 (s, 3H). ^{13}C NMR (101 MHz, DMSO) δ 165.3, 158.8, 152.4, 149.6, 144.4, 139.6, 137.3, 127.8, 127.6, 127.6, 123.1, 121.0, 114.0, 99.3, 56.6, 55.3, 53.7, 31.1, 18.3. LCMS: 487.4.

2.11. (1-(4-(Trifluoromethyl)phenyl)-1*H*-1,2,3-triazol-4-yl)methyl 4-(3-methoxyphenyl)-6-methyl-2-oxo-1,2,3,4-tetrahydropyrimidine-5-carboxylate (3k)

IR (KBr) ν/cm^{-1} 3414, 2964, 2349, 1718, 1642, 1333, 843, 788, 726. ^{1}H NMR (400 MHz, DMSO) δ 9.25 (s, 1H), 8.74 (s, 1H), 8.10 (d, *J* = 8.5 Hz, 2H), 7.97 (d, *J* = 8.6 Hz, 2H), 7.72 (s, 1H), 7.15 (t, *J* = 7.9 Hz, 1H), 6.82 – 6.65 (m, 3H), 5.19 – 5.11 (m, 3H), 3.59 (s, 3H), 2.24 (s, 3H). ^{13}C NMR (101 MHz, DMSO) δ 165.3, 159.6, 152.5, 150.0, 146.6, 144.3, 130.0, 127.7, 127.6, 123.2, 121.0, 118.6, 112.8, 112.6, 98.9, 56.7, 55.3, 54.1, 18.3. LCMS: 487.3.

2.12. (1-(4-(Trifluoromethyl)phenyl)-1*H*-1,2,3-triazol-4-yl)methyl 4-(4-ethoxyphenyl)-6-methyl-2-oxo-1,2,3,4-tetrahydropyrimidine-5-carboxylate (3l)

IR (KBr) ν/cm^{-1} 3323, 2982, 2350, 1696, 1644, 1331, 839, 769, 696. ^{1}H NMR (400 MHz, DMSO) δ 9.20 (s, 1H), 8.65 (s, 1H), 8.09 (d, *J* = 8.5 Hz, 2H), 7.96 (d, *J* = 8.6 Hz, 2H), 7.65 (s, 1H), 7.05 (d, *J* = 8.6 Hz, 2H), 6.72 (d, *J* = 8.6 Hz, 2H), 5.26 – 5.05 (m, 3H), 3.88 – 3.72 (m, 2H), 2.23 (s, 3H), 1.19 (t, *J* = 7.0 Hz, 3H).^{13}C NMR (101 MHz, DMSO) δ 165.3, 158.1, 152.4, 149.5, 144.4, 139.6, 137.2, 127.8, 127.6, 127.6, 123.0, 121.0, 114.5, 99.3, 63.3, 56.5, 53.7, 18.3, 14.9. LCMS: 501.6.

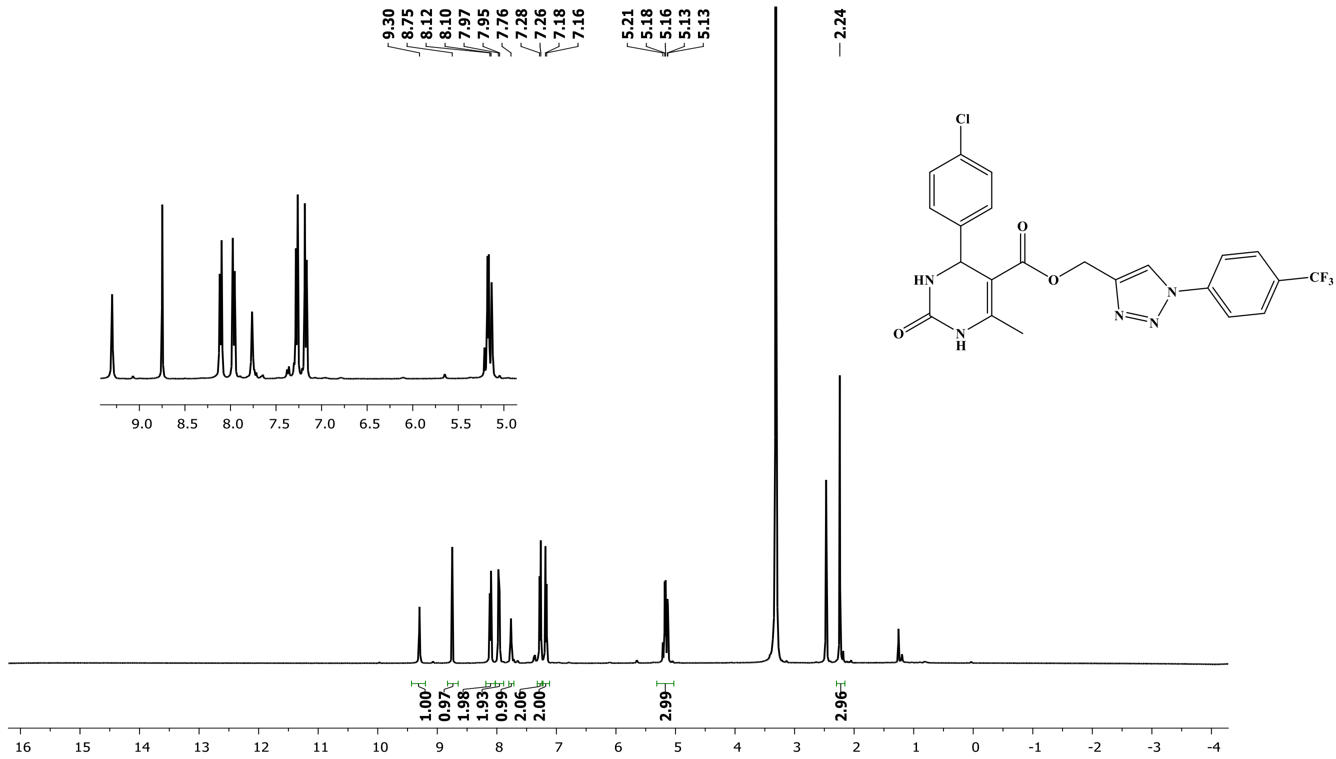

Figure S1: ^{1}H-NMR of (1-(4-(trifluoromethyl)phenyl)-1H-1,2,3-triazol-4-yl)methyl 4-(4-chlorophenyl)-6-methyl-2-oxo-1,2,3,4-tetrahydropyrimidine-5-carboxylate (**3a**).

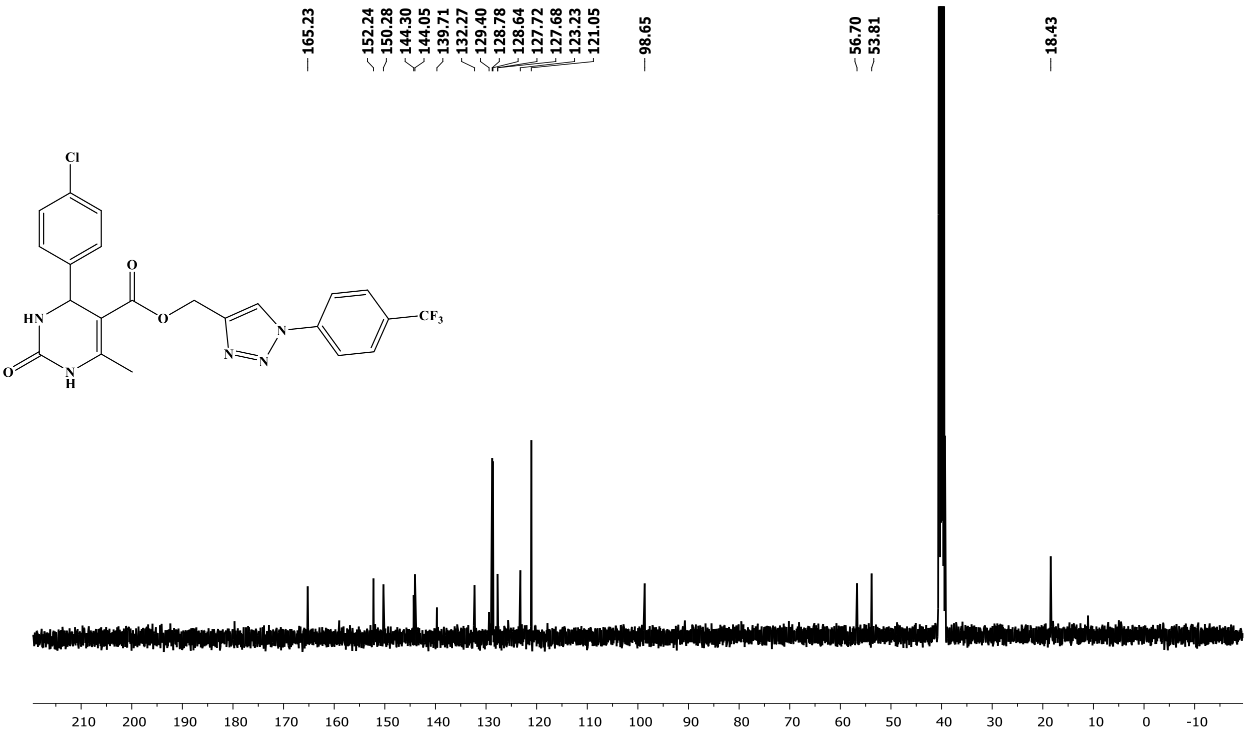

Figure S2: ^{13}C-NMR of (1-(4-(trifluoromethyl)phenyl)-1H-1,2,3-triazol-4-yl)methyl 4-(4-chlorophenyl)-6-methyl-2-oxo-1,2,3,4-tetrahydropyrimidine-5-carboxylate (**3a**)

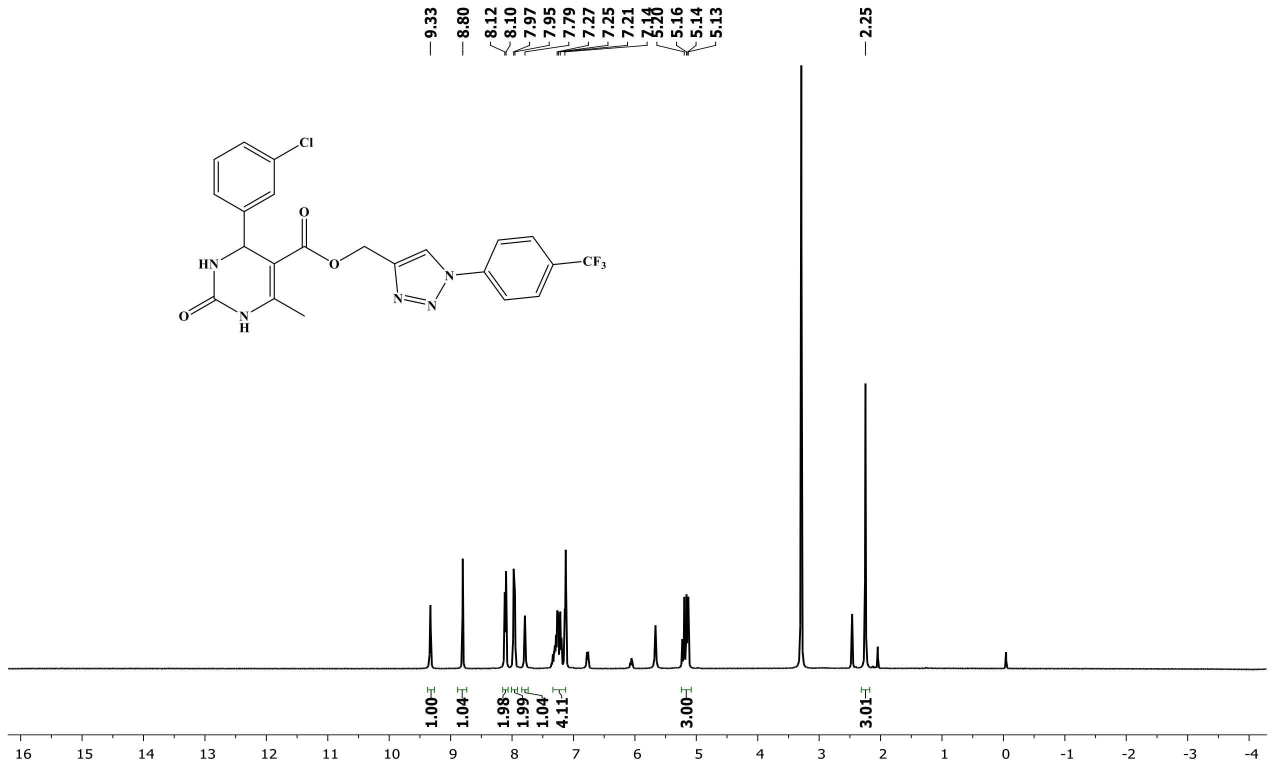

Figure S3: ^{1}H-NMR of (1-(4-(trifluoromethyl)phenyl)-1H-1,2,3-triazol-4-yl)methyl 4-(3-chlorophenyl)-6-methyl-2-oxo-1,2,3,4-tetrahydropyrimidine-5-carboxylate **(3b)**

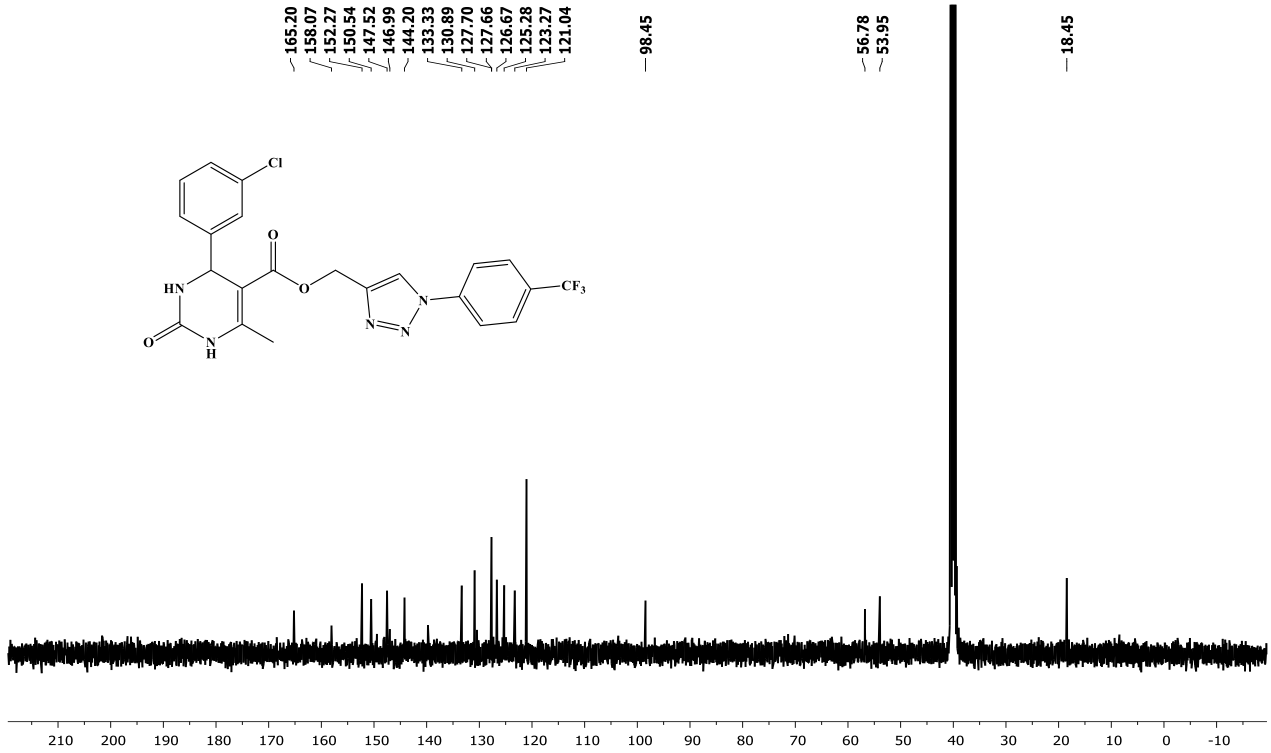

Figure S4: ^{13}C-NMR of (1-(4-(trifluoromethyl)phenyl)-1H-1,2,3-triazol-4-yl)methyl 4-(3-chlorophenyl)-6-methyl-2-oxo-1,2,3,4-tetrahydropyrimidine-5-carboxylate **(3b)**

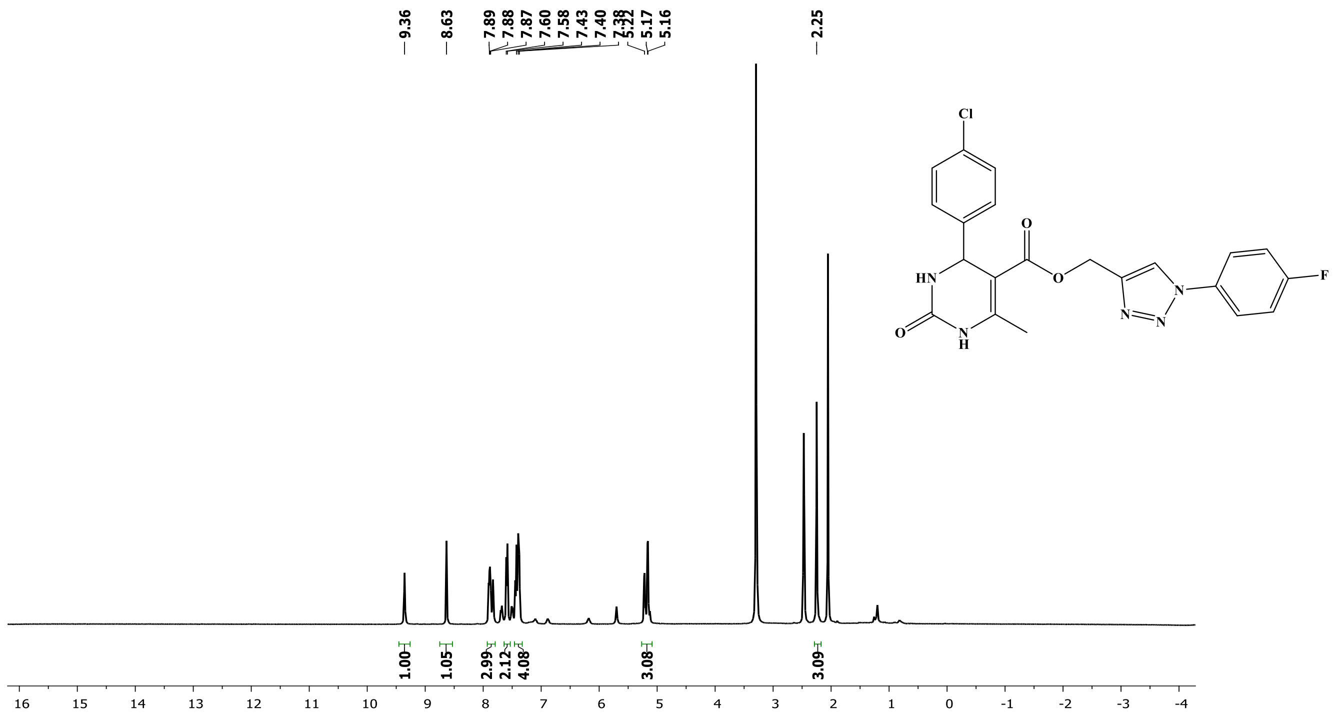

Figure S5: ^{1}H-NMR of (1-(4-fluorophenyl)-1H-1,2,3-triazol-4-yl)methyl 4-(4-chlorophenyl)-6-methyl-2-oxo-1,2,3,4-tetrahydropyrimidine-5-carboxylate **(3c)**

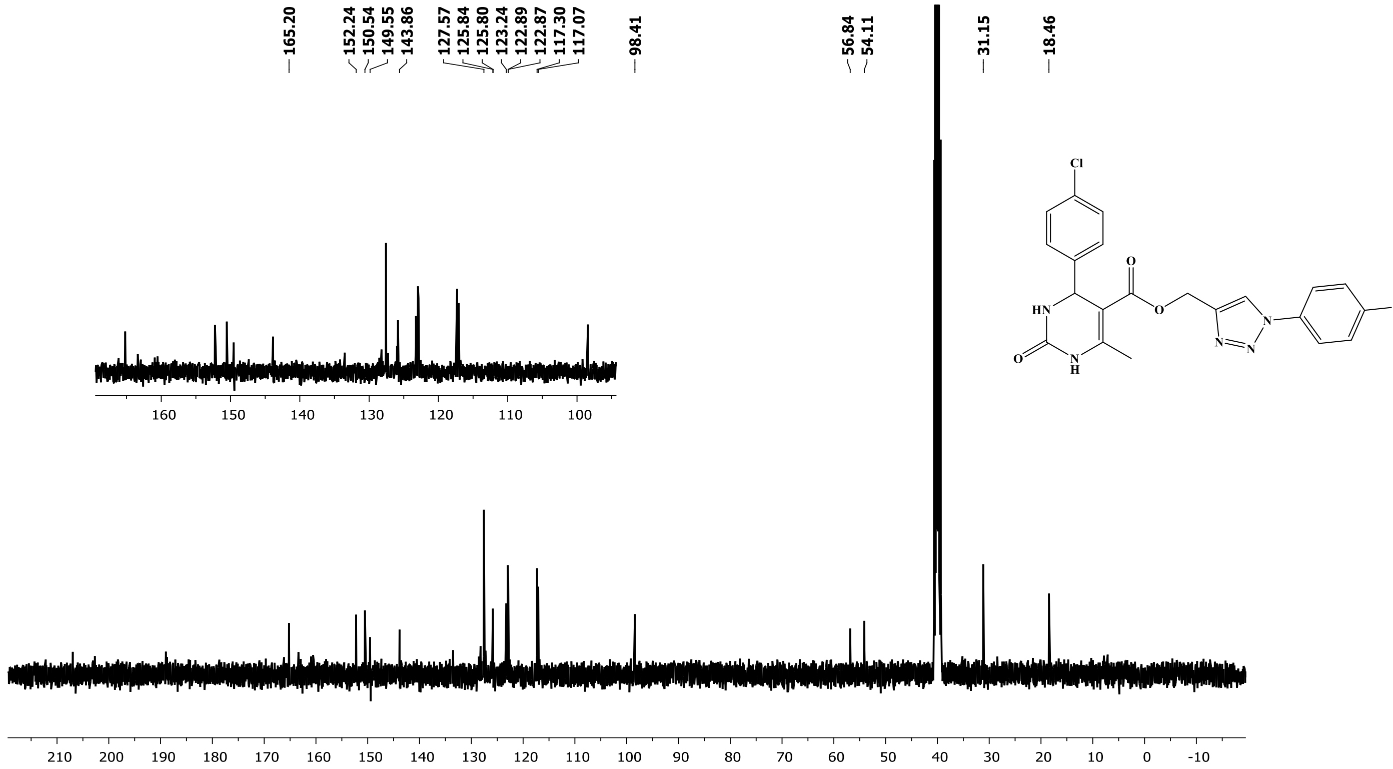

Figure S6: ^{13}C-NMR of (1-(4-fluorophenyl)-1H-1,2,3-triazol-4-yl)methyl 4-(4-chlorophenyl)-6-methyl-2-oxo-1,2,3,4-tetrahydropyrimidine-5-carboxylate **(3c)**

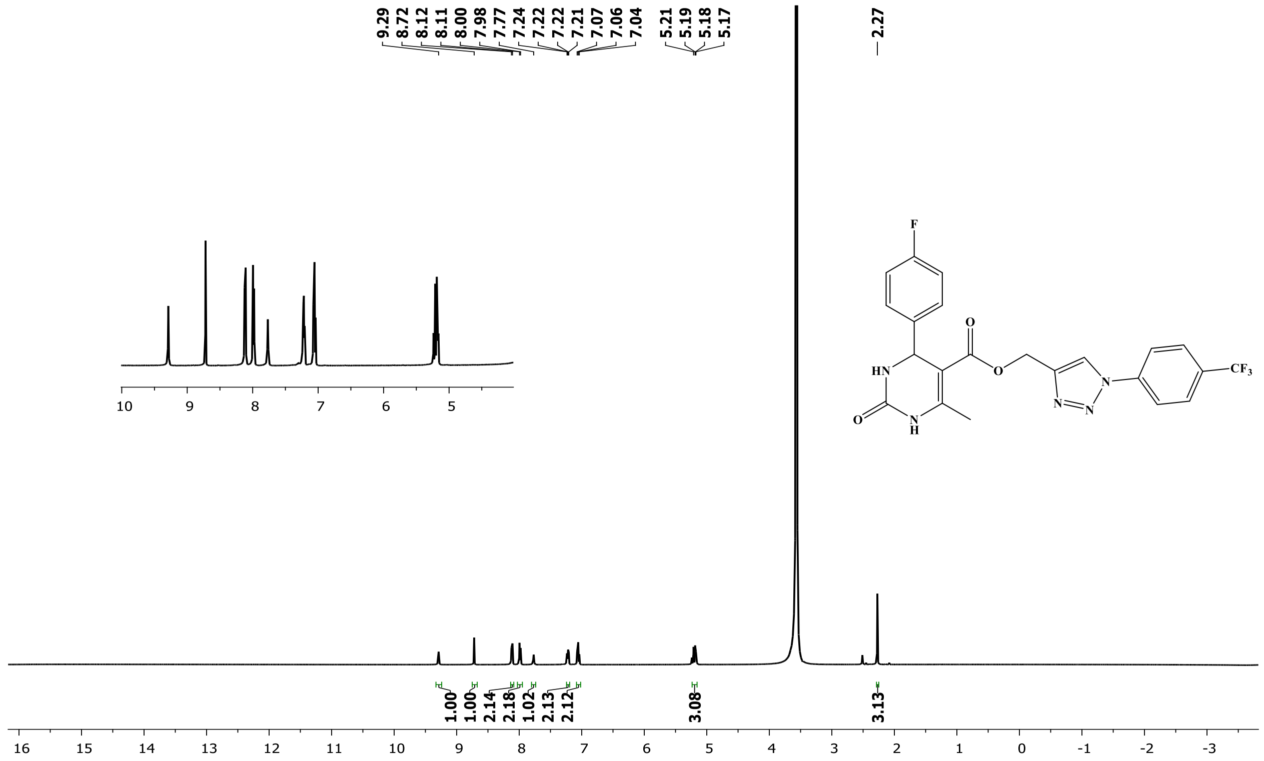

Figure S7: ^{1}H-NMR of (1-(4-(trifluoromethyl)phenyl)-1H-1,2,3-triazol-4-yl)methyl 4-(4-fluorophenyl)-6-methyl-2-oxo-1,2,3,4-tetrahydropyrimidine-5-carboxylate **(3d)**

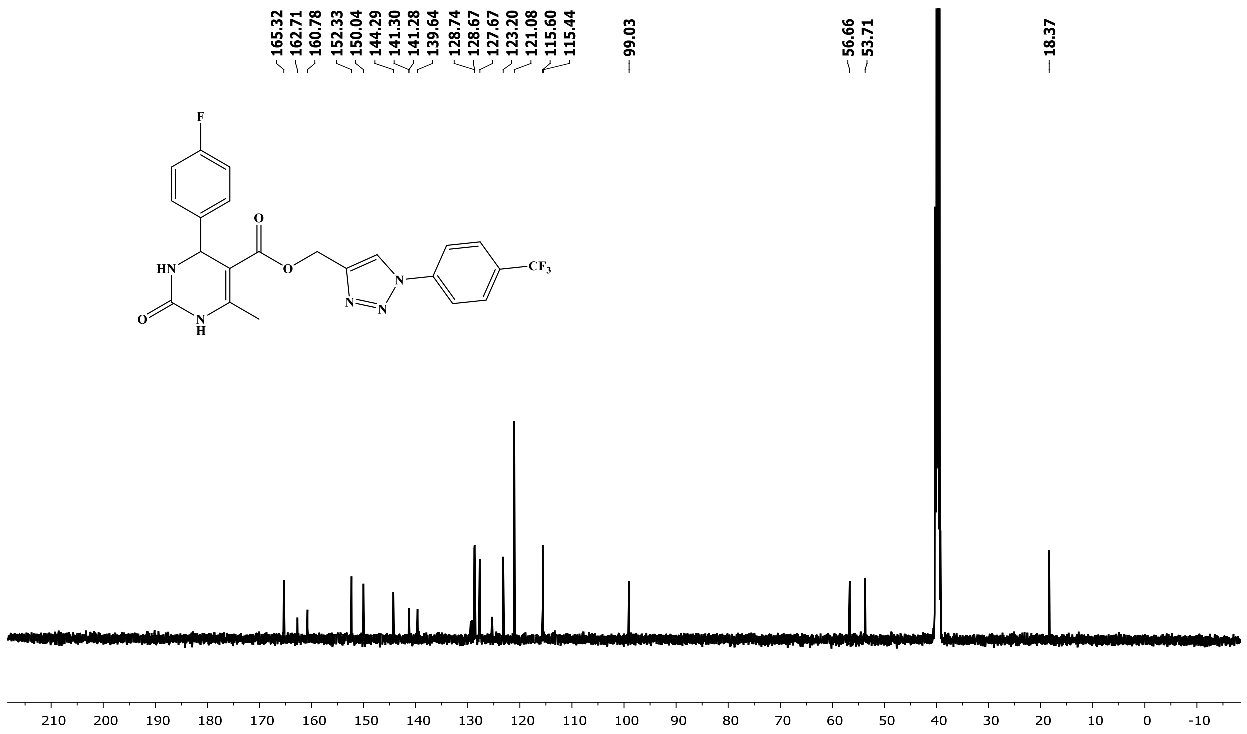

Figure S8: ^{13}C-NMR of (1-(4-(trifluoromethyl)phenyl)-1H-1,2,3-triazol-4-yl)methyl 4-(4-fluorophenyl)-6-methyl-2-oxo-1,2,3,4-tetrahydropyrimidine-5-carboxylate **(3d)**

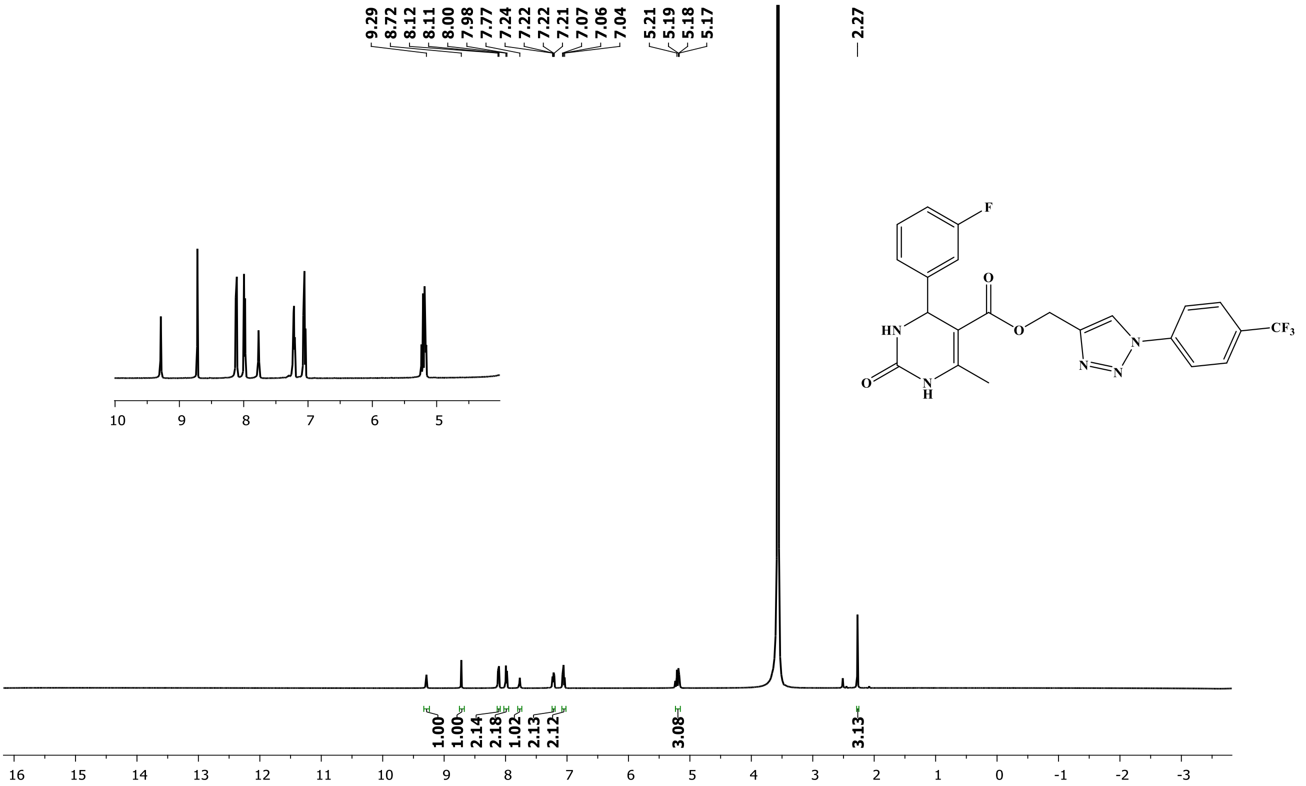

Figure S9: ^{1}H-NMR of (1-(4-(trifluoromethyl)phenyl)-1H-1,2,3-triazol-4-yl)methyl 4-(3-fluorophenyl)-6-methyl-2-oxo-1,2,3,4-tetrahydropyrimidine-5-carboxylate **(3e)**

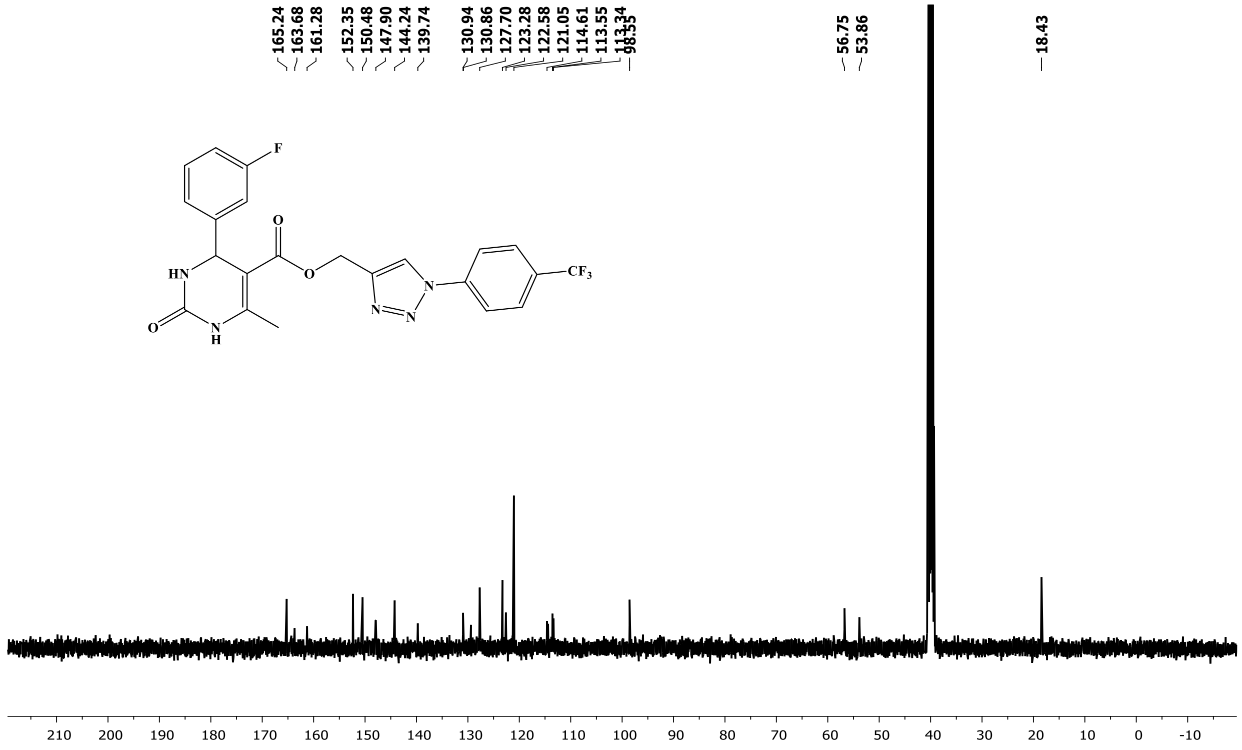

Figure S10: ^{13}C-NMR of (1-(4-(trifluoromethyl)phenyl)-1H-1,2,3-triazol-4-yl)methyl 4-(3-fluorophenyl)-6-methyl-2-oxo-1,2,3,4-tetrahydropyrimidine-5-carboxylate **(3e)**

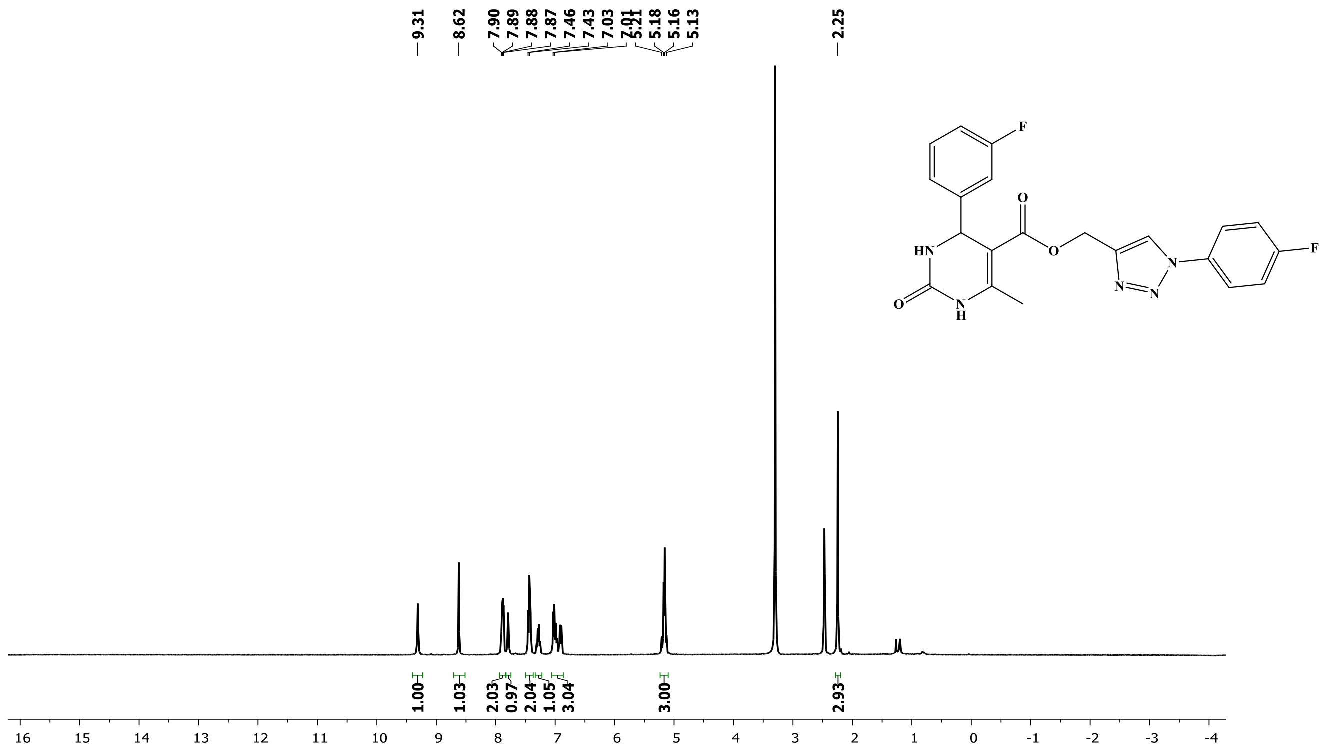

Figure S11: ^{1}H-NMR of (1-(4-fluorophenyl)-1H-1,2,3-triazol-4-yl)methyl 4-(3-fluorophenyl)-6-methyl-2-oxo-1,2,3,4-tetrahydropyrimidine-5-carboxylate **(3f)**

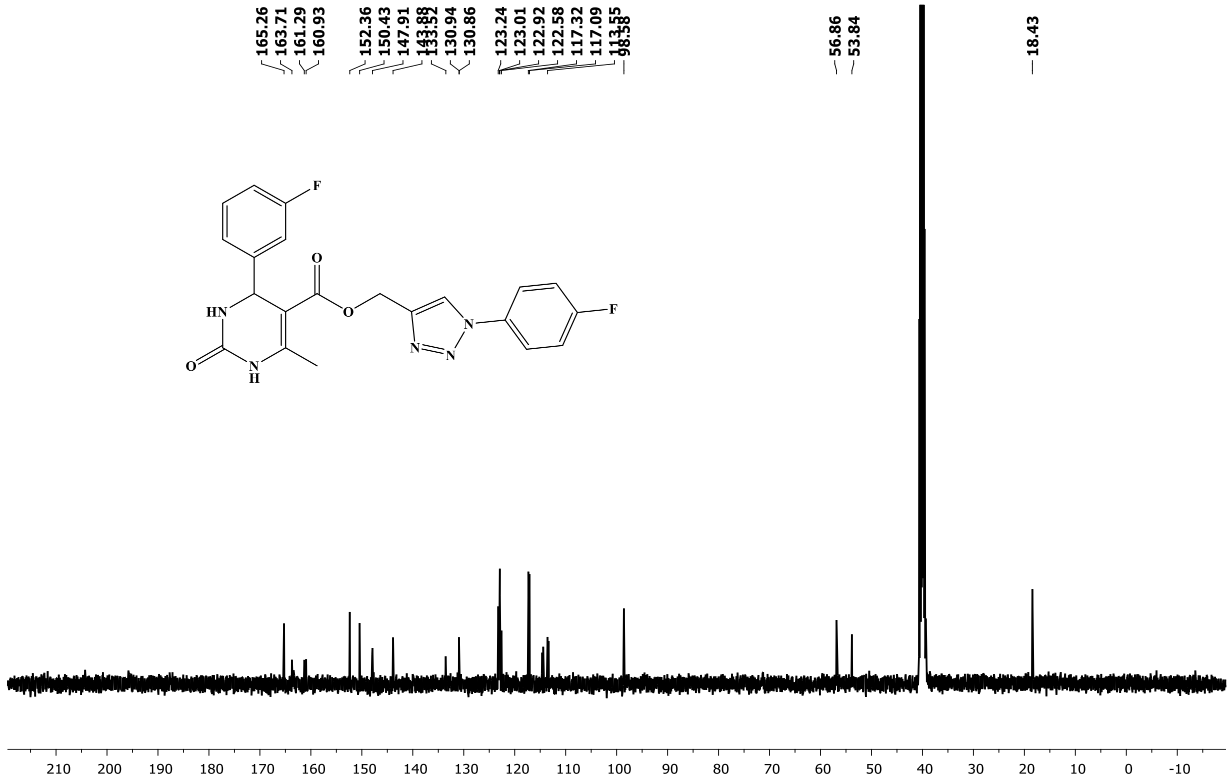

Figure S12: ^{13}C-NMR of (1-(4-fluorophenyl)-1H-1,2,3-triazol-4-yl)methyl 4-(3-fluorophenyl)-6-methyl-2-oxo-1,2,3,4-tetrahydropyrimidine-5-carboxylate **(3f)**

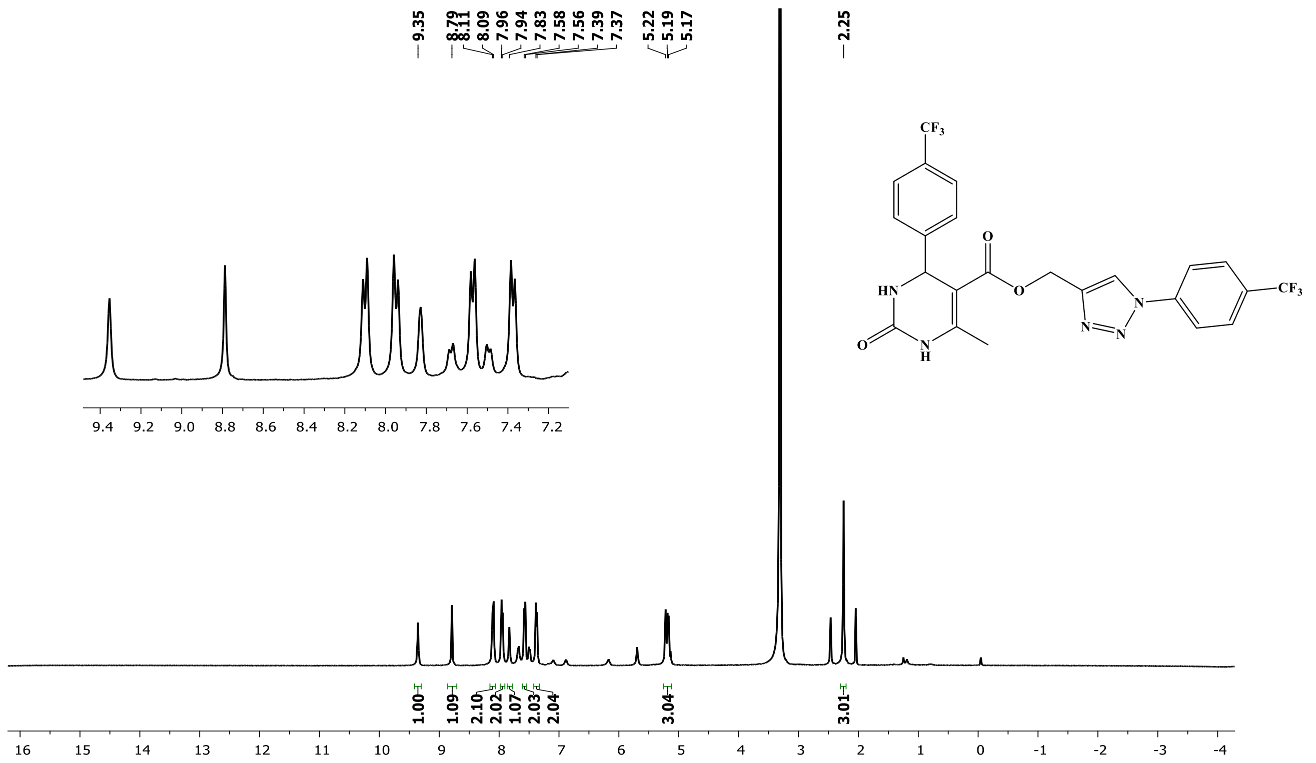

Figure S13: ^{1}H-NMR of (1-(4-(trifluoromethyl)phenyl)-1H-1,2,3-triazol-4-yl)methyl 6-methyl-2-oxo-4-(4-(trifluoromethyl)phenyl)-1,2,3,4-tetrahydropyrimidine-5-carboxylate **(3g)**

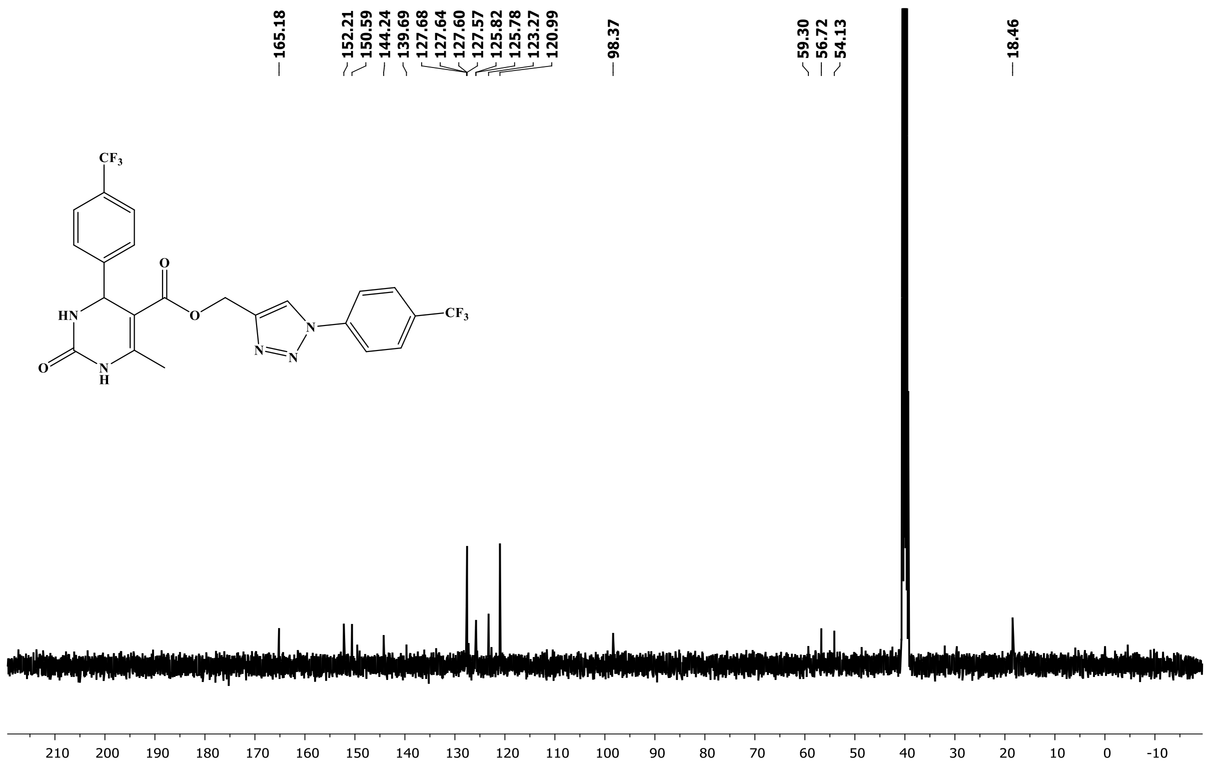

Figure S14: ^{13}C-NMR of (1-(4-(trifluoromethyl)phenyl)-1H-1,2,3-triazol-4-yl)methyl 6-methyl-2-oxo-4-(4-(trifluoromethyl)phenyl)-1,2,3,4-tetrahydropyrimidine-5-carboxylate **(3g)**

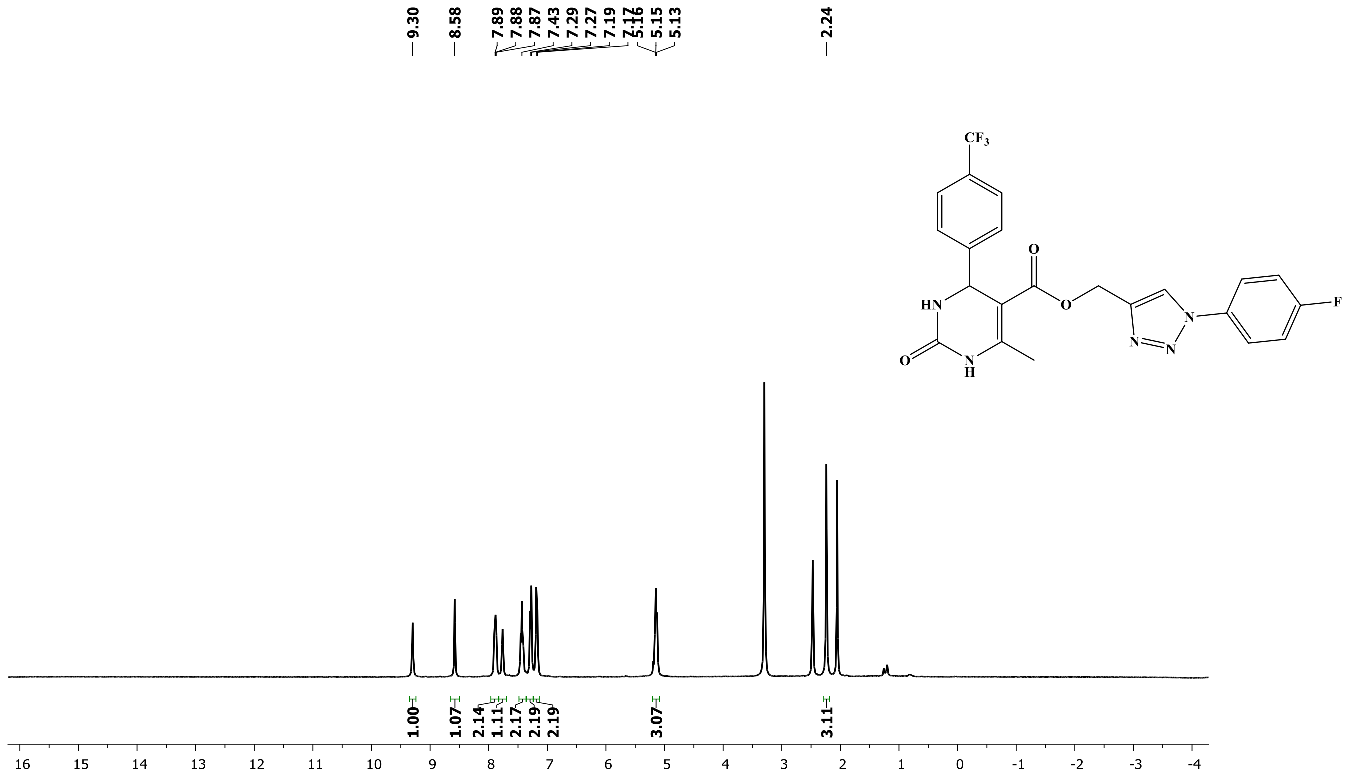

Figure S15: ^{1}H-NMR of (1-(4-fluorophenyl)-1H-1,2,3-triazol-4-yl)methyl 6-methyl-2-oxo-4-(4-(trifluoromethyl)phenyl)-1,2,3,4-tetrahydropyrimidine-5-carboxylate **(3h)**

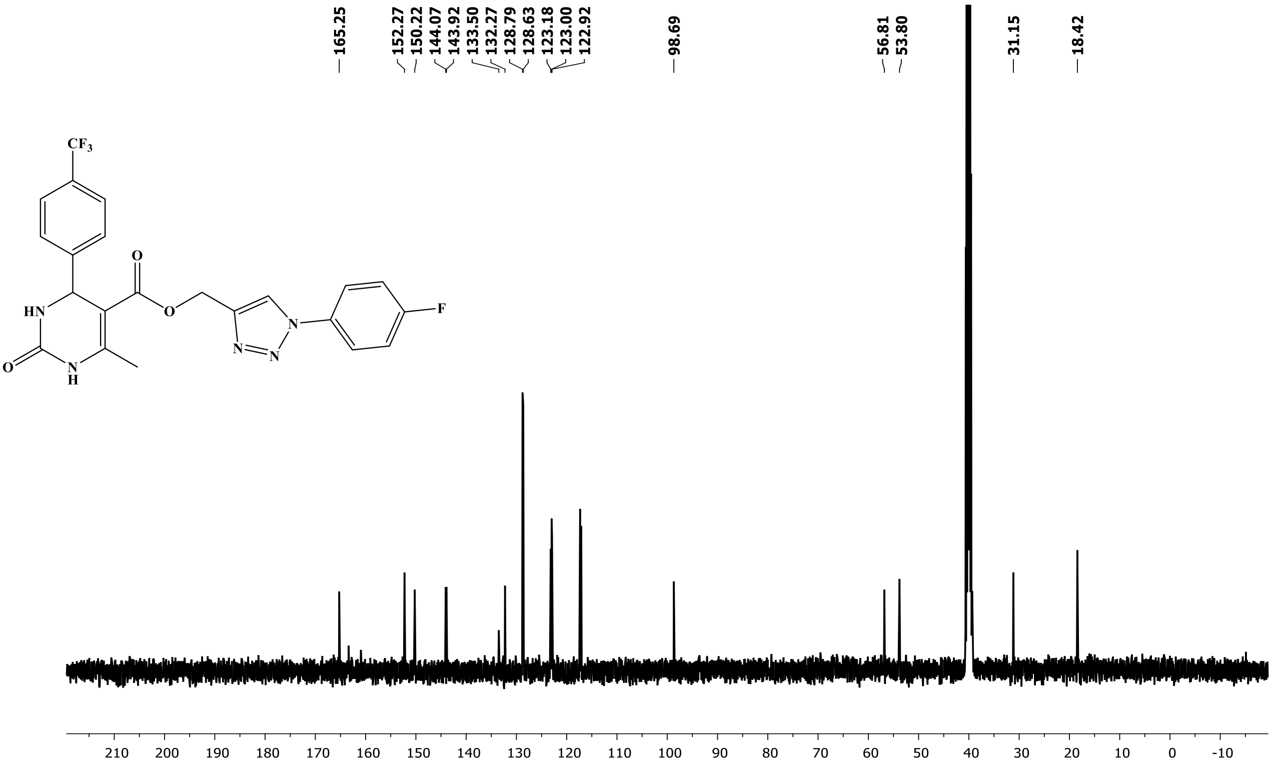

Figure S16: ^{13}C-NMR of (1-(4-fluorophenyl)-1H-1,2,3-triazol-4-yl)methyl 6-methyl-2-oxo-4-(4-(trifluoromethyl)phenyl)-1,2,3,4-tetrahydropyrimidine-5-carboxylate **(3h)**

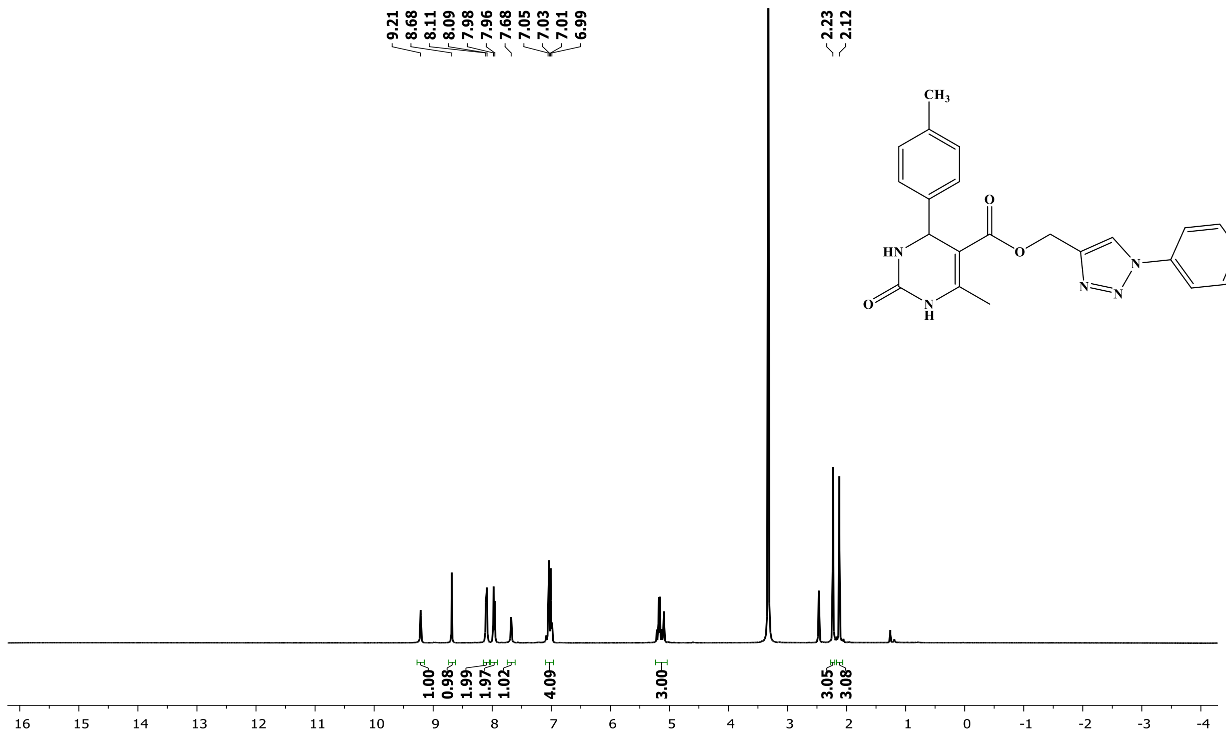

Figure S17: ^{1}H-NMR of (1-(4-(trifluoromethyl)phenyl)-1H-1,2,3-triazol-4-yl)methyl 6-methyl-2-oxo-4-(p-tolyl)-1,2,3,4-tetrahydropyrimidine-5-carboxylate **(3i)**

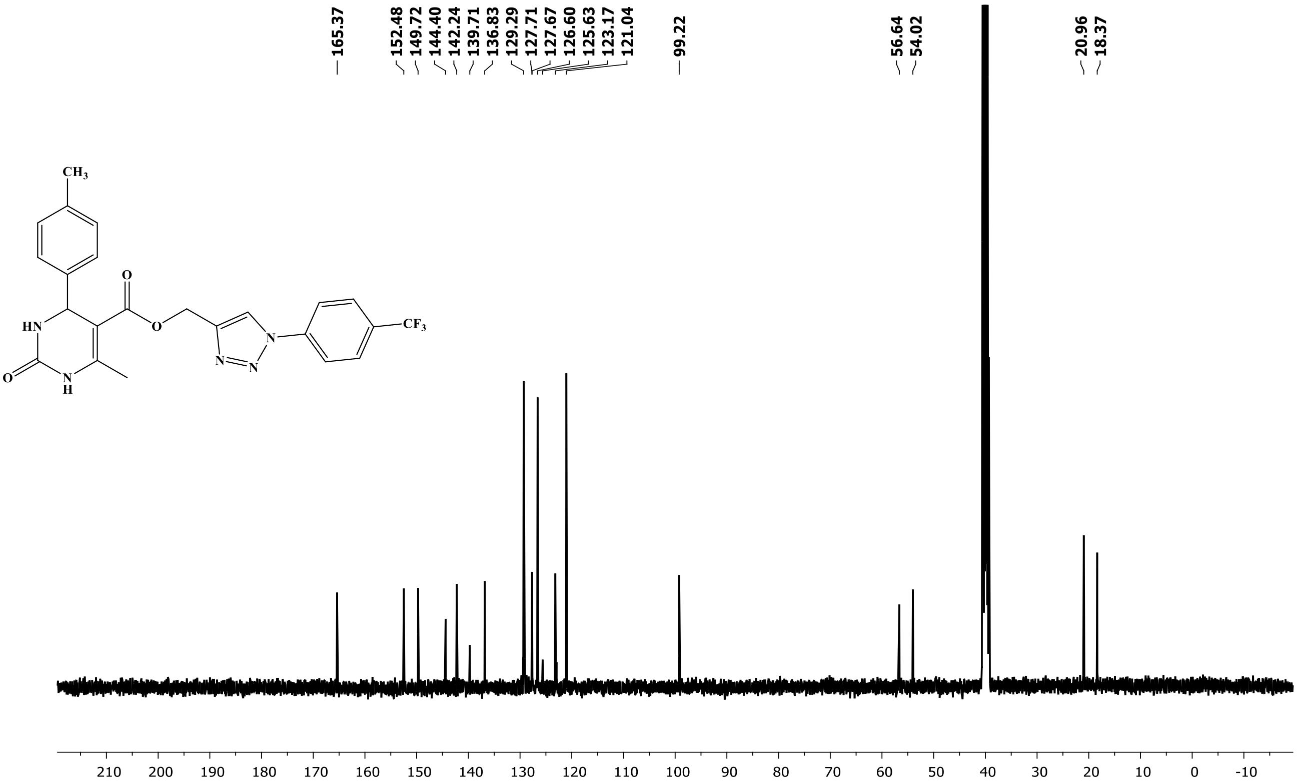

Figure S18: ^{13}C-NMR of (1-(4-(trifluoromethyl)phenyl)-1H-1,2,3-triazol-4-yl)methyl 6-methyl-2-oxo-4-(p-tolyl)-1,2,3,4-tetrahydropyrimidine-5-carboxylate **(3i)**

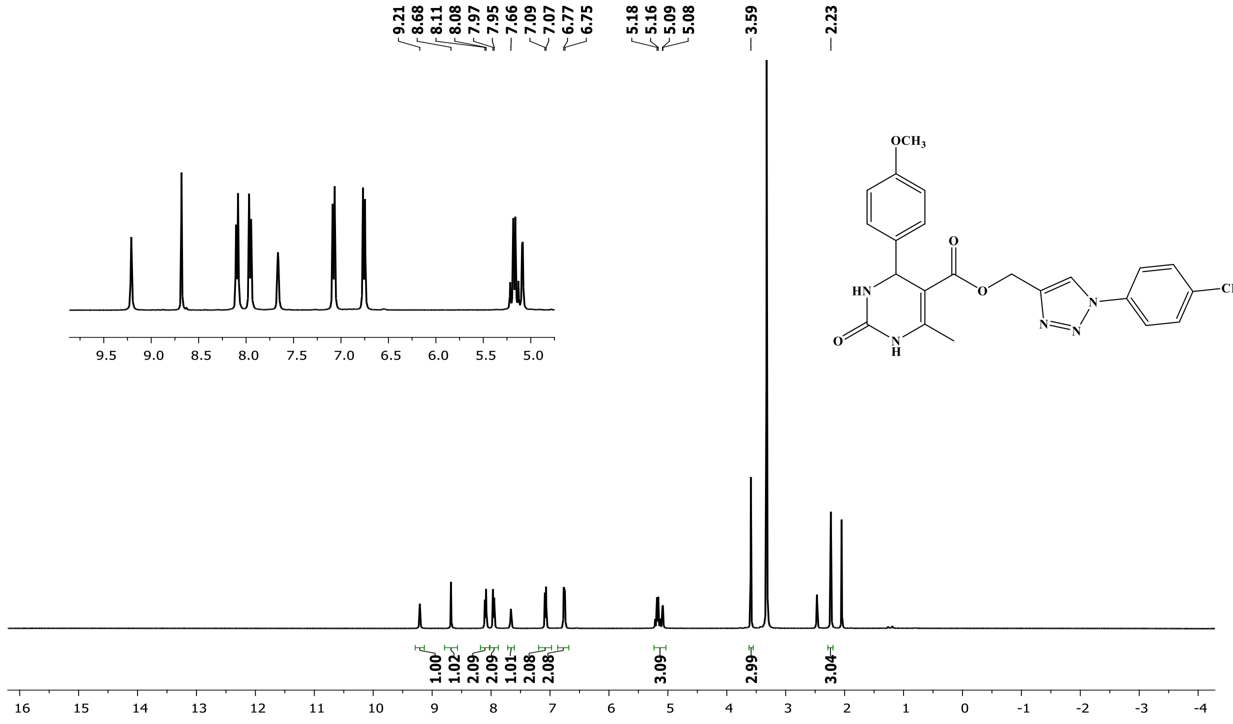

Figure S19: ^{1}H-NMR of (1-(4-(trifluoromethyl)phenyl)-1H-1,2,3-triazol-4-yl)methyl 4-(4-methoxyphenyl)-6-methyl-2-oxo-1,2,3,4-tetrahydropyrimidine-5-carboxylate **(3j)**

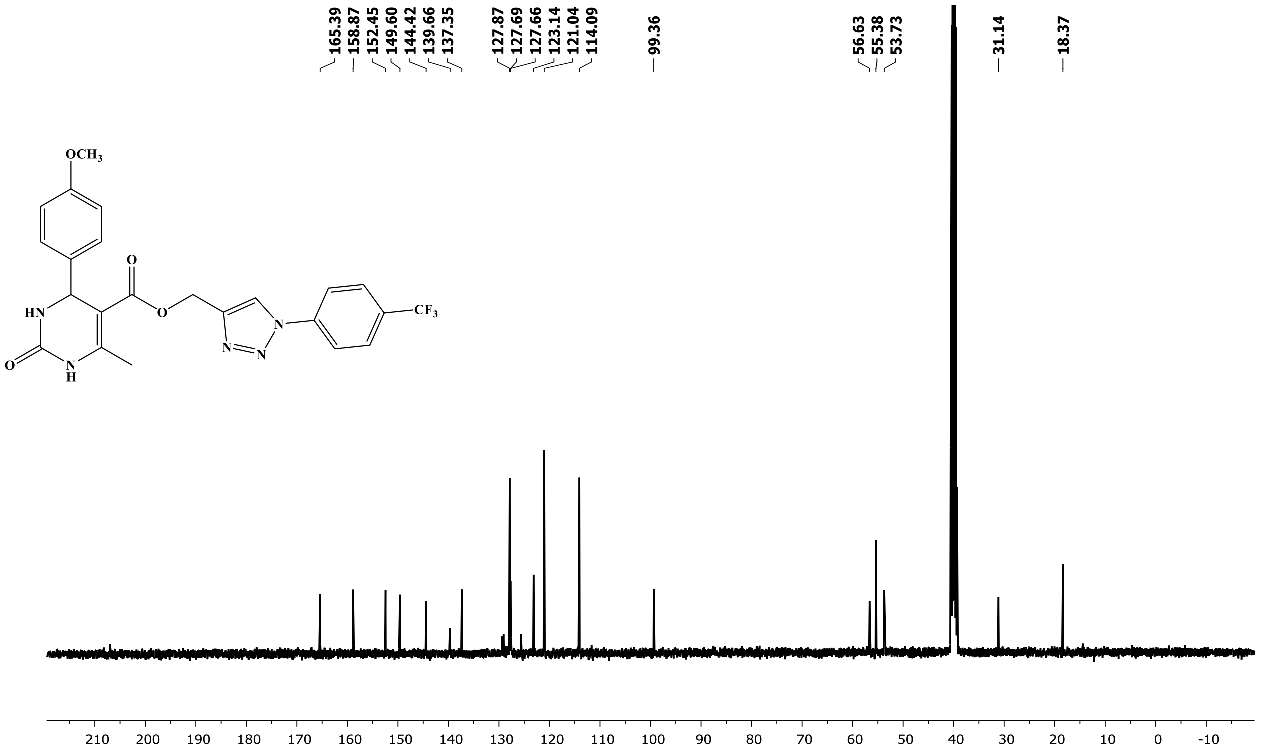

Figure S20: ^{13}C-NMR of (1-(4-(trifluoromethyl)phenyl)-1H-1,2,3-triazol-4-yl)methyl 4-(4-methoxyphenyl)-6-methyl-2-oxo-1,2,3,4-tetrahydropyrimidine-5-carboxylate **(3j)**

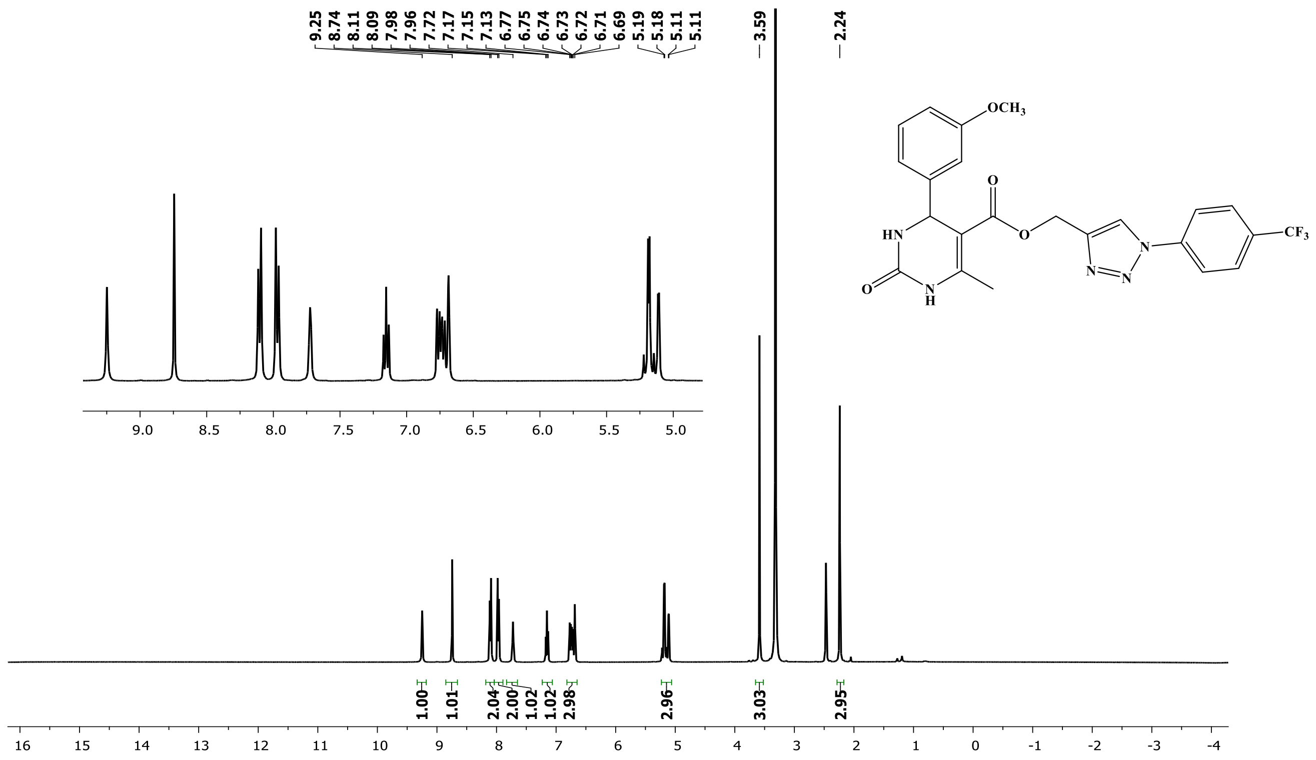

Figure S21: ^{1}H-NMR of (1-(4-(trifluoromethyl)phenyl)-1H-1,2,3-triazol-4-yl)methyl 4-(3-methoxyphenyl)-6-methyl-2-oxo-1,2,3,4-tetrahydropyrimidine-5-carboxylate **(3k)**

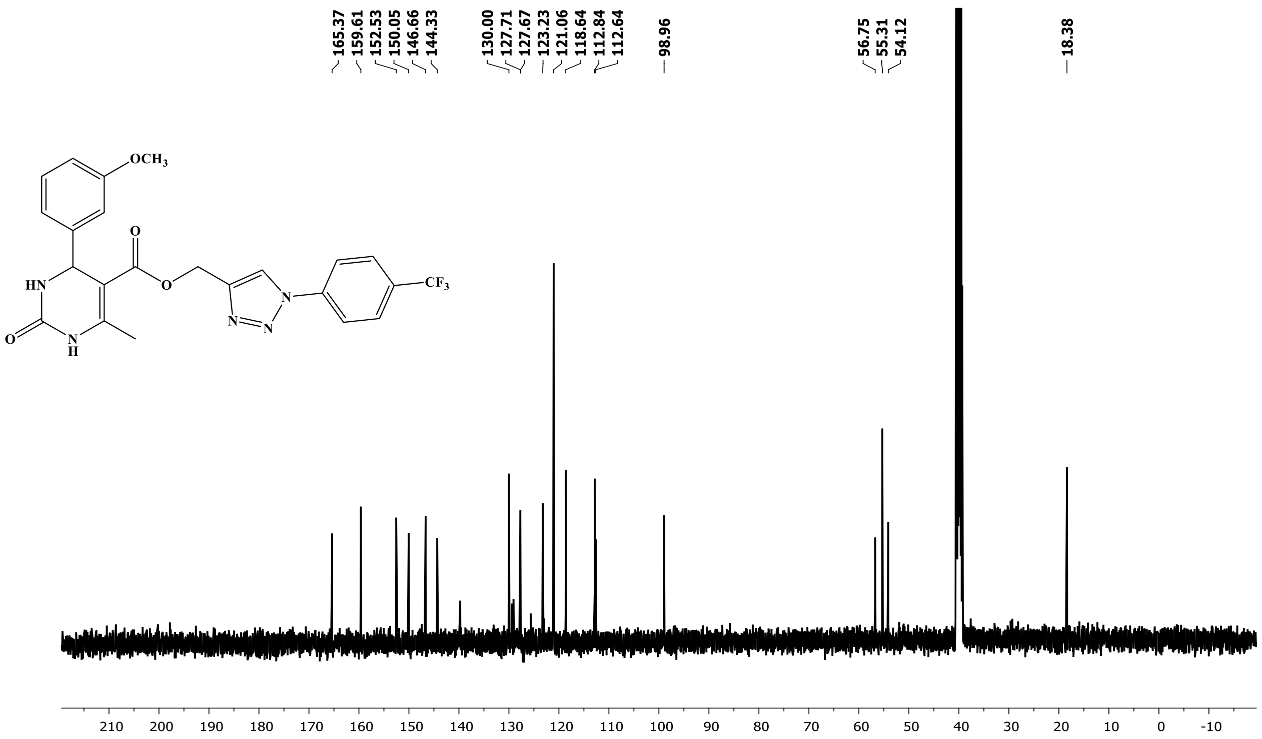

Figure S22: ^{13}C-NMR of (1-(4-(trifluoromethyl)phenyl)-1H-1,2,3-triazol-4-yl)methyl 4-(3-methoxyphenyl)-6-methyl-2-oxo-1,2,3,4-tetrahydropyrimidine-5-carboxylate **(3k)**

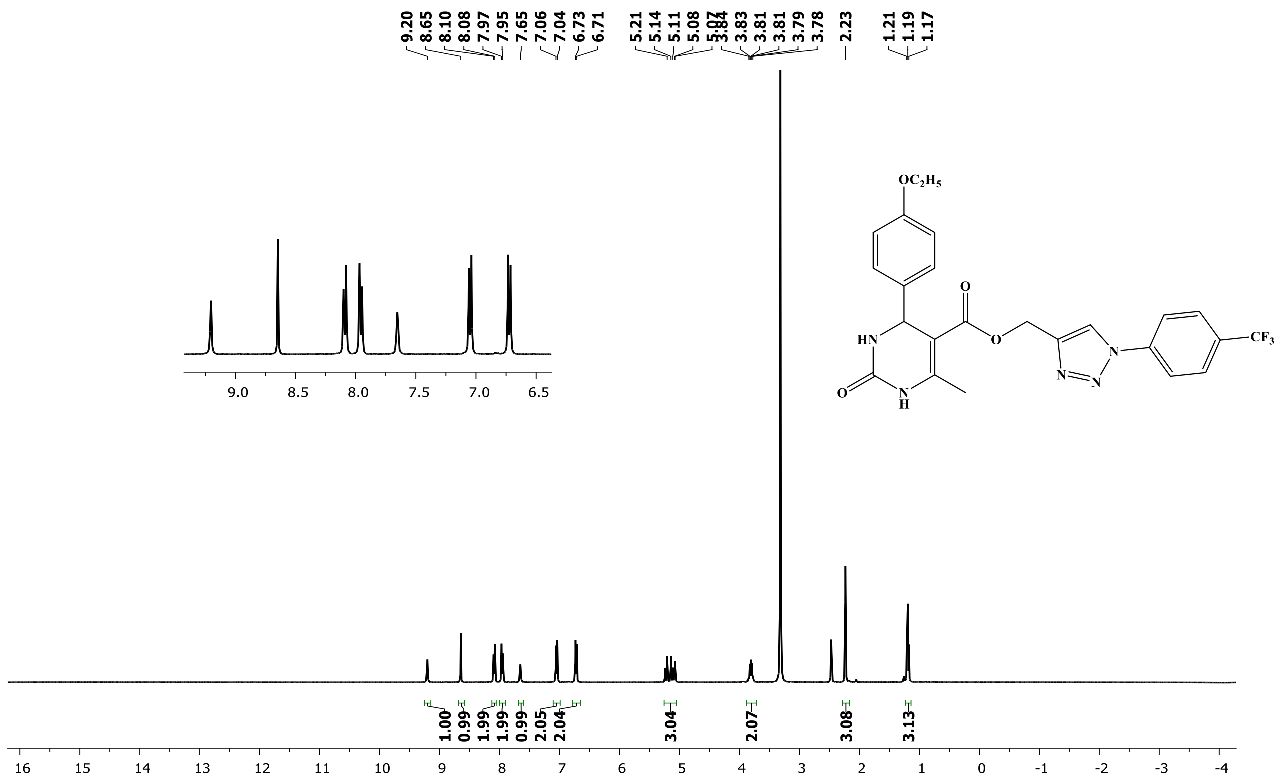

Figure S23: ^{1}H-NMR of (1-(4-(trifluoromethyl)phenyl)-1H-1,2,3-triazol-4-yl)methyl 4-(4-ethoxyphenyl)-6-methyl-2-oxo-1,2,3,4-tetrahydropyrimidine-5-carboxylate **(3l)**

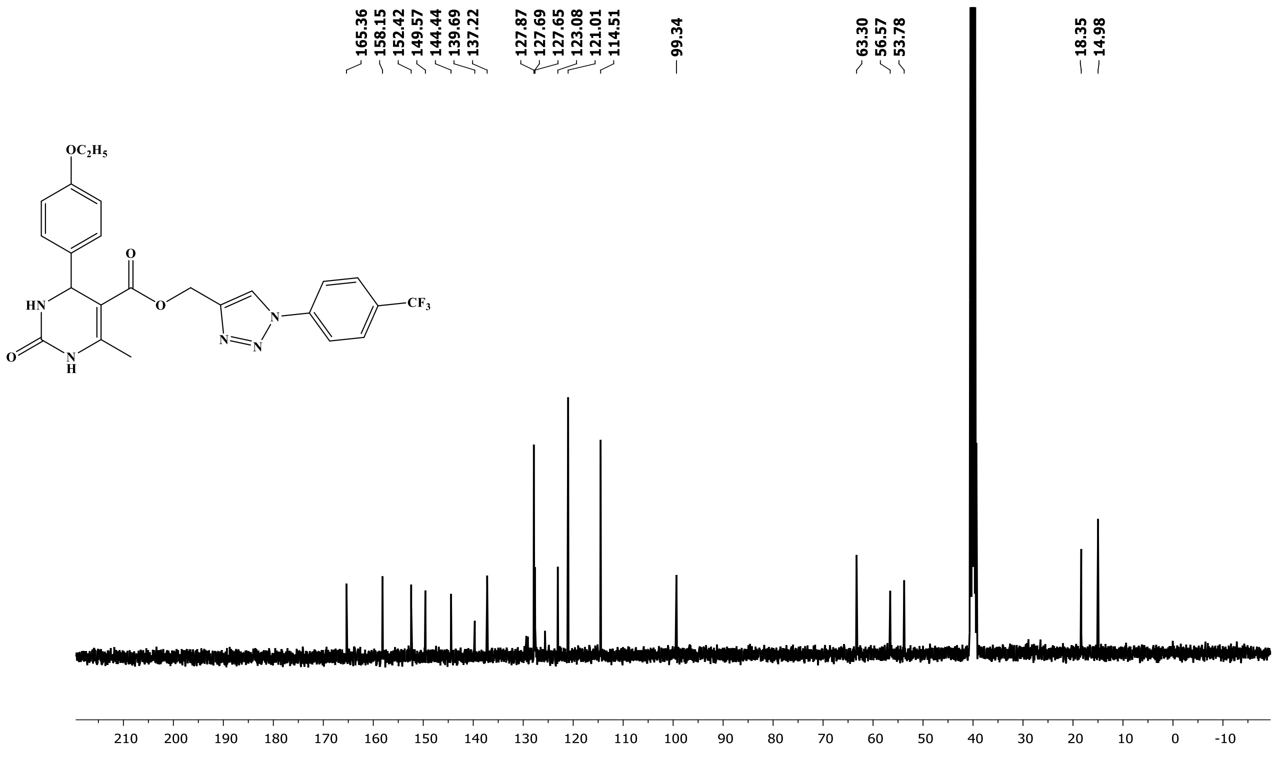

Figure S24: ^{13}C-NMR of (1-(4-(trifluoromethyl)phenyl)-1H-1,2,3-triazol-4-yl)methyl 4-(4-ethoxyphenyl)-6-methyl-2-oxo-1,2,3,4-tetrahydropyrimidine-5-carboxylate **(3l)**
